# Insights Into Infections and Inflammation in Prostate Cancer Development and Management: An Overview

**DOI:** 10.1155/bmri/7584584

**Published:** 2026-08-07

**Authors:** Ensiyeh Bahadoran, Abouzar Babaei, Shahla Shahbazi, Milad Badri, Farhad Nikkhahi, Samira Sabzi

**Affiliations:** ^1^ Cellular and Molecular Research Center, Research Institute for Prevention of Non-Communicable Disease, Qazvin University of Medical Sciences, Qazvin, Iran, qums.ac.ir; ^2^ Medical Microbiology Research Center, Qazvin University of Medical Sciences, Qazvin, Iran, qums.ac.ir; ^3^ Infectious Diseases Research Center, Health Policy and Promotion Institute, Kermanshah University of Medical Sciences, Kermanshah, Iran, kums.ac.ir

**Keywords:** benign prostatic hyperplasia (BPH), chronic inflammation, prostate cancer, prostate microbiota, prostatitis, sexually transmitted infections (STIs), urinary tract infections (UTIs)

## Abstract

Prostate cancer (PCa) represents a major public health concern and continues to be one of the leading causes of cancer‐related mortality among men worldwide. Current epidemiological projections suggest that the incidence and associated burden of PCa are likely to increase, emphasizing the importance of advancing preventive measures, improving early diagnostic approaches, and refining targeted therapeutic strategies. While established risk factors, including age, genetic predisposition, lifestyle‐related factors, ethnicity, and androgen signaling, have been extensively studied, these factors alone do not fully explain the observed patterns of PCa development. Increasing evidence suggests that infection‐associated chronic inflammation plays a central role in prostate tumor initiation and progression. Chronic inflammation of the prostate, arising from conditions such as prostatitis, benign prostatic hyperplasia (BPH), lower urinary tract infections (UTIs), sexually transmitted infections (STIs), and prolonged or repeated catheterization, has been associated with histological and molecular changes that may predispose prostate tissue to malignant transformation. Moreover, emerging evidence suggests that alterations in the prostate microbiome may sustain inflammatory signaling, thereby influencing tumor initiation and progression. This review synthesizes current epidemiological findings and experimental evidence linking infection‐associated inflammation to the development of PCa. Particular focus is placed on inflammatory signaling pathways implicated in prostate tumorigenesis, including nuclear factor kappa B (NF‐*κ*B), signal transducer and activator of transcription 3 (STAT3), cyclooxygenase‐2/prostaglandin E_2_ (COX‐2/PGE_2_), interleukin‐6 and interleukin‐8 signaling, Toll‐like receptor (TLR) pathways, and activation of the NLRP3 inflammasome. In addition, we discuss therapeutic strategies that are aimed at modulating these pathways and their potential relevance in PCa management. A more comprehensive understanding of the interactions between infection, chronic inflammation, and PCa development may facilitate the identification of novel biomarkers and therapeutic targets. Such advances could ultimately contribute to improved risk stratification, earlier detection, and more effective treatment of PCa.

## 1. Introduction

Prostate cancer (PCa) represents 7.3% of new cancer cases reported across 118 countries, ranking as the second most prevalent type of cancer. In 2020, it was responsible for around 370,000 deaths globally, making it the fifth leading cause of cancer‐related fatalities among men [1, 2]. In the United States, PCa is the second leading cause of mortality after lung cancer [3]. With an increasingly aging population and economic growth, the prevalence of PCa is expected to increase, presenting a considerable challenge in healthcare management [4, 5]. PCa is estimated to grow from 1.4 million new cases annually in 2020 to 2.9 million by 2040. Significant increases in the disease are anticipated as a result of shifting age patterns and increasing life expectancies [6]. Despite notable treatment advancements in recent years, the mortality rate from PCa has shown minimal improvement over the past decade in the United Kingdom and several other nations. In England, approximately half of the PCa cases are diagnosed at Stage 3 or 4 [7]. Understanding the risk factors associated with PCa development, as well as exploring their potential correlations with other health conditions, is crucial for early detection, intervention, and treatment strategies [8].

The prostate gland plays a crucial role in male reproductive health and can be influenced by several conditions, including prostatitis, benign prostatic hyperplasia (BPH), and PCa [9]. Urinary tract infections (UTIs) are common bacterial infections that affect individuals of all ages worldwide [10]. Lower UTIs (LUTIs) typically occur in the bladder and urethra, and there has been growing interest in how UTIs may impact the prostate gland and the development of PCa [11]. The correlation between LUTI and PCa remains a fascinating area of research, with studies focusing on early detection methods, the effects on quality of life, and possible connections to the progression of the disease [11].

Recent research has focused on the connection between the microbiome, inflammation, and the development of PCa, emphasizing how imbalances in microbial communities may contribute to the disease′s progression [12, 13]. Moreover, sexually transmitted diseases (STDs) and frequent sexual encounters with sex workers are other infectious etiologies that are positively associated with PCa [14].

The common underlying factor in all of these infections is inflammation [15–18]. Chronic inflammation has been linked to many cancer stages, including cellular transformation, promotion, proliferation, angiogenesis, metastasis, invasion, and survival [19]. Anti‐inflammatory treatments are effective in the prevention and treatment of cancer [20].

Given the growing burden of this disease, it is necessary to understand how specific infections can influence prostate carcinogenesis. This comprehensive review is aimed at exploring the relationship between infections and PCa development and management. The key mentioned in this review consists of risk factors, such as prostatitis, BPH, LUTI, prostate microbiome, and STDs. We also highlight inflammatory signaling pathways that contribute to PCa progression. This insight will help us better understand the potential role of infections in PCa development, identify possible preventive strategies, and explore novel therapeutic approaches that target infection‐related inflammation and tumor progression.

## 2. Method/Search Strategy

This review is a narrative review of the literature. The search was conducted across multiple databases, including Google Scholar, PubMed, Scopus, and Web of Science, up to August 2025. Keywords and medical subject headings terms such as “prostate cancer,” “prostatitis,” “benign prostatic hyperplasia,” “urinary tract infection,” “sexually transmitted infection,” “prostate microbiome,” “catheterization,” “inflammation,” and “signaling pathways” were used in various combinations. Additional references were identified from the bibliographies of relevant articles. Both clinical and preclinical studies, including observational, experimental, and mechanistic investigations, were considered. Articles were included if they addressed the role of infections or inflammation in the initiation, progression, or treatment of PCa.

## 3. Understanding Prostate and PCa

The primary role of the prostate is to produce semen, a fluid rich in nutrients that supports and carries sperm during ejaculation. The urethra, which is the tube that carries urine from the bladder to the outside of the body, runs through the prostate. This connection is why issues with the prostate can impact both urinary and reproductive health [21].

PCa generally originates from the cells in the prostate. It often starts in the glandular cells lining the prostate ducts, where normal cells can mutate and begin to grow unchecked. Over time, these cancer cells may form tumors in the prostate [22]. PCa can be classified into five categories based on its origin. The most common type is adenocarcinoma, but metastasis can occur much more rapidly in other forms. These subtypes consist of acinar adenocarcinoma, ductal adenocarcinoma, transitional cell (or urothelial) cancer, squamous cell cancer, and small‐cell PCa [23]. Failure to treat it can lead to the spread of PCa from the prostate to other parts of the body, such as the bones, lymph nodes, or nearby organs [24].

Several risk factors can increase the rate of PCa, including age (risk increases with age), family history of PCa, genetic mutations (e.g., BRCA genes), race (African American men have a higher risk), and certain lifestyle factors [25, 26]. In addition, altered androgen metabolism is another important factor [27]. Modifiable risk factors for PCa include smoking, diet, physical activity, the use of certain medications, and occupational exposures [2]. Additionally, numerous studies over the last decade have highlighted the importance of both innate and adaptive immune systems, along with the surrounding microenvironment [28, 29].

PCa might not show any noticeable symptoms in its early stages. As the condition progresses, it can lead to changes in urination, such as needing to go frequently and having trouble starting or stopping urination, as well as issues with erections, discomfort, back pain, and unexplained weight loss [30, 31]. It is essential for individuals to promptly consult a healthcare provider if they experience any concerning symptoms. Early detection through regular screening plays a critical role in improving treatment outcomes and survival rates [32].

**Figure 1:**
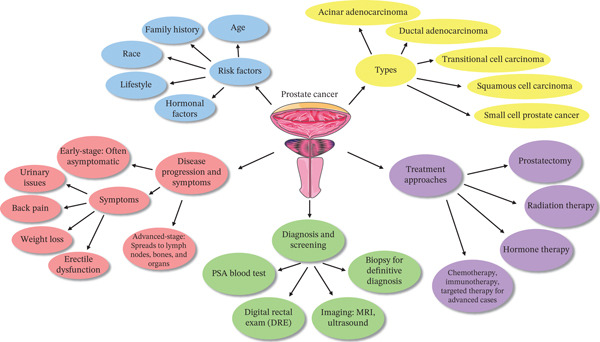
Overview of PCa, including its risk factors, types, disease progression and symptoms, diagnosis and screening methods, and treatment approaches.

Treatment options for PCa vary based on several factors, such as the stage and grade of cancer, as well as the patient′s overall health. Common approaches include active surveillance for low‐risk cancers, surgery (like prostatectomy), hormone therapy, radiation therapy (RT), chemotherapy, immunotherapy, and targeted therapy [33]. While many therapies are associated with high cure rates, a considerable number of PCa patients experience a recurrence of the disease within 10 years [34]. Figure 1 provides a comprehensive overview of PCa, covering its risk factors, types, symptoms, diagnosis, and treatment options. Despite global efforts to provide early detection methods and innovative treatment options, the advanced stages of this disease are untreatable.

## 4. Infectious Factors and PCa

### 4.1. Prostatitis

Prostatitis is an inflammation or swelling of the prostate gland, affecting 35%–50% of men during their lifetime [35]. Urologists see more patients with prostatitis than with BPH or PCa [36]. There are four subcategories of prostatitis: Category I is acute bacterial prostatitis; Category II is chronic bacterial prostatitis; Category III is chronic prostatitis/chronic pelvic pain syndromes (CP/CPPSs); and Category IV is asymptomatic prostatitis [37]. CP/CPPS is the most prevalent type of prostatitis, accounting for 90%–95% of all diagnoses. It is estimated that up to 15% of men have this condition [38]. Although it is frequently challenging to detect and treat, its symptoms include discomfort and lower urinary tract problems. Research is currently underway to determine its precise pathogenesis [39]. In contrast to BPH and PCa, which primarily affect older men, prostatitis can affect individuals of any age [40].

Prostatitis has been included in the list of risk factors for PCa, although there are controversies. It has been shown that 61.53% of PCa patients also had prostatitis, suggesting a possible link between prostatic infections and PCa development. Moreover, in patients who underwent radical prostatectomy (RP), a significant relationship was found between prostatitis and low‐risk PCa [41]. These findings demonstrate that early detection and treatment of prostatitis could be important for reducing PCa risk or progression [41]. Jiang et al. evaluated 20 case‐control studies and found a strong positive correlation between prostatitis and PCa. However, the strength of the association varies according to study design (personal interview vs. clinical), time, and geographic location [42]. According to a study conducted in a large, multiracial, and ethnic cohort as part of the California Men′s Health Study (CMHS), a history of prostatitis and prolonged symptoms of prostatitis are associated with an increased risk of PCa [43]. Jung et al. showed that prostatitis is linked to a higher prevalence of PCa. However, compared to chronic prostatitis, acute prostatitis is linked to a higher incidence of PCa [44]. A history of clinical chronic prostatitis can also considerably increase the incidence of PCa in the general population, according to a meta‐analysis of 15 case‐control studies; however, it is unclear if this is true for African Americans [45].

In contrast, some studies have found a negative correlation between prostatitis and PCa, or linked just a subset of prostatitis to PCa. Although a history of clinical prostatitis was statistically linked to PCa in earlier meta‐analyses, this association was significantly reduced and nonsignificant when detection bias was taken into account, according to a systematic review and meta‐analysis of 38 studies. A strong inverse trend was observed between the degree of methodological rigor in accounting for detection bias and the strength of the association, suggesting that many of the previously reported positive associations were due to increased PCa screening triggered by prostatitis diagnosis. Stratified analyses showed that differential recall bias might also contribute to the observed associations, although to a lesser extent than detection bias [46]. Moreover, a potential association was observed between prostatitis and PCa, particularly with acute bacterial prostatitis, which showed the highest risk increase, with an average interval of 12.2 years between the last episode and cancer diagnosis. Chronic bacterial prostatitis exhibited a weaker but still notable association, whereas CPPS was not linked to PCa [47]. Chronic inflammatory infiltration (CII) was reported to be a strong negative independent predictor of PCa detection in a group of individuals who had a prostate initial biopsy, suggesting that its presence was linked to a decreased risk of PCa. CII Type IV should be regarded as an adjunctive parameter in rebiopsy or active surveillance procedures [48].

In a study investigating the mechanism by which prostatic inflammation influences PCa, it was found that prostatic inflammation increases TNF‐*α*, IL‐1*β*, IL‐6, and TGF‐*β* levels in prostatitis and PCa compared with BPH. In an inflammatory environment, both primary prostate epithelial cells and PCa cells show increased viability, androgen receptor (AR) expression, AR‐mediated transcription, and epithelial–mesenchymal transition (EMT) processes. Conversely, nuclear factor kappa B (NF‐*κ*B) inhibition reduced these effects [49].

Although numerous studies suggest an association between prostatitis and increased PCa risk, the evidence remains inconsistent due to potential detection and recall biases. Mechanistic studies support a biological link by showing that chronic inflammation can promote pathways involved in prostate carcinogenesis. However, current data are insufficient to establish a causal relationship, and further well‐designed prospective studies are needed to clarify the role of prostatitis in PCa development.

### 4.2. BPH

BPH occurs due to abnormally hyperplastic proliferation of the fibromuscular and epithelial tissues of the transition zone and periurethral area [50]. The prevalence of this illness increases with age and is more prevalent among older males [51]. In this regard, research indicates that over 70% of men between the ages of 60 and 69 years have BPH, while over 90% of men over the age of 70 have pathological BPH [52]. Changes in hormones, inflammation, growth factors, cell receptor signaling, epigenetics, nutrition, exercise, and prostate microbiota all have an impact on this illness and cause cellular proliferation [53]. Lower urinary tract symptoms (LUTSs) caused by BPH comprise voiding symptoms (intermittency, hesitancy, poor stream, terminal dribble, and straining) and storage issues (urgency, nocturia, and increased frequency), which adversely affect the quality of life of patients and their families [53].

The prevalence of BPH and prostatitis is high in Europe, North America, and Asia, ranging from 3% to 16% [54]. Regarding the relationship between prostatitis and BPH, a higher chance of BPH‐related events was linked to prostatitis [41, 55]. Furthermore, inflammatory aspects were found in 43.1% of BPH cases, predominantly as chronic inflammation. The occurrence of chronic inflammation significantly increased with prostate volume but varied according to prostate size [56]. It was shown that COX‐2 is upregulated in prostate luminal epithelial cells, particularly in atrophic lesions, such as postatrophic hyperplasia and simple atrophy. COX‐2 expression was associated with increased cell proliferation, apoptotic resistance via Bcl‐2 overexpression, and the presence of inflammatory cells, mainly T lymphocytes and macrophages [57]. It has also been shown that tumor necrosis factor receptor‐associated factor 6 (TRAF6) signaling stimulates BPH stromal cell proliferation through Akt/mTOR activation [58]. Chronic inflammation may contribute to BPH pathogenesis by infiltrating immune cells, particularly B lymphocytes, T lymphocytes, and macrophages, which participate in disease progression. The study found that elevated levels of proinflammatory cytokines (TNF‐*α* and IL‐1*β*) were reduced by Permixon treatment, which correlated with an improvement in urinary symptoms [59]. In the evaluation of 282 patients treated surgically for complicated and/or symptomatic BPH, prostatic inflammation was found to be common. Then, 81% of the patients showed T lymphocyte infiltration (CD3+), 52% showed B lymphocytes (CD20+), and 82% showed macrophages (CD163+). High‐grade inflammation was significantly associated with increased prostate volume [60].

The symptoms of BPH and PCa are frequently mistaken, especially in the early stages, and are sometimes underdiagnosed [61]. Moreover, the phenotypic link between BPH and PCa can be explained by their comparable inherited genetic profile [62].

Some epidemiologic studies have found a positive correlation between both BPH and prostatitis and PCa [54, 63]. The largest epidemiological investigation to date to evaluate the risk of PCa after receiving a BPH diagnosis has been conducted in Danish males who were monitored for up to 27 years, and it has been shown that clinical BPH was associated with a two‐ to eight‐fold higher likelihood of PCa death and a two‐ to three‐fold increased risk of PCa incidence [64]. Moreover, among Korean patients, BPH and/or prostatitis are associated with a higher incidence of PCa. It was the highest in the combination of prostatitis and BPH in particular, which is probably linked to effects comparable to those of PSA screening [63]. In a meta‐analysis of 42 studies, Zhang et al. demonstrated that BPH or prostatitis may increase the risk of PCa. Meanwhile, those who have experienced prostatitis in the past may be at a higher risk of developing BPH [54]. Using data from Taiwan′s National Health Insurance, researchers found that BPH had a higher odds ratio for PCa than prostatitis. Men with both conditions had an even greater odds ratio [65]. Interestingly, shared genetic anomalies have been introduced between BPH and PCa, including SRDAR2, CYP17, CYP19, 14‐3‐3‐sigma, RASSF1A, MT1G, and MDR1 [66].

However, in a study of 5068 men, there was no association between symptomatic BPH and PCa risk, regardless of BPH definition, detection method, or cancer grade [67]. Moreover, men with BPH were found to have little to no overall increased risk of PCa compared with the general population, with only a 2% excess incidence after 10 years. However, the risk varied according to treatment type. For example, after the first 5 years, patients treated with transvesicular adenomectomy had a 22% lower incidence and 23% lower mortality, whereas those treated with transurethral resection of the prostate (TURP) experienced a 10% higher incidence but 17% lower mortality. In contrast, after the first 5 years, patients with BPH who did not undergo surgical intervention had significantly increased risks, with an 18% higher incidence and 77% higher mortality from prostate carcinoma [68].

Metabolites of serum organic acids may be employed as noninvasive indicators to differentiate between PCa, BPH, and prostatitis. Key metabolites, including citric acid, pyroglutamic acid, and phenylacetic acid, showed high diagnostic accuracy. Additionally, pyroglutamic acid, ethylmalonic acid, and 5‐hydromethyl‐2‐furoic acid helped to differentiate PCa patients with PSA < 4.0 ng/mL from those with PSA > 4.0 ng/mL [69].

Current evidence suggests that BPH and PCa share several biological features, including chronic inflammation, overlapping molecular pathways, and common genetic alterations. Although multiple epidemiological studies report an increased risk of PCa among men with BPH, the findings remain inconsistent and may be influenced by detection bias and differences in clinical management. Therefore, BPH should be considered a potential marker of increased PCa susceptibility rather than a confirmed independent risk factor, warranting further investigation.

### 4.3. LUTIs

UTI is a common bacterial infection that affects any component of the urinary tract, including the kidneys, ureters, bladder, and urethra [70, 71]. In 2019, the number of UTI cases worldwide exceeded 400 million, representing a 2.4‐fold increase since 1990. This led to approximately 235,000 deaths and five million disabilities [72]. UTIs are primarily triggered by uropathogenic *Escherichia coli* (UPEC) bacteria, responsible for 75% of uncomplicated UTIs and 65% of complicated UTIs [73]. Recent multistate studies have found that *Enterococcus faecium*, *Staphylococcus aureus*, *Acinetobacter baumannii*, *Pseudomonas aeruginosa*, and *Enterobacter* species, collectively known as ESKAPE pathogens, are frequently identified as causative agents of healthcare‐associated UTIs following *E.coli* [74–76]. UTIs with antibiotic‐resistant bacterial strains pose a major challenge to successful treatment [71]. Various factors can increase the risk of developing UTI, including poor hygiene practices, urinary catheter use, sexual activity, hormonal changes, urinary tract abnormalities, and underlying health conditions that weaken the immune system [77]. LUTI, like urethritis and cystitis, has been proposed to potentially lead to prostate infection and inflammation (prostatitis) and contribute to the onset of PCa [78]. It may be challenging to eliminate bacteria that have roots in the prostate due to the restricted penetration of antibiotic drugs into the gland or the existence of prostate stones that harbor bacterial colonies [79, 80]. Men experiencing LUTS might have storage symptoms such as nocturia, frequency, and urgency, as well as voiding symptoms such as hesitancy, intermittency, and a weak stream [81]. All symptoms of LUTS among men increased in prevalence with advancing age, especially in men aged > 60 years [82]. Numerous studies on LUTS and PCa have mostly described LUTS‐related quality of life in PCa patients and the impact of LUTS on PCa screening [83, 84]. One study examined the impact of LUTS on the progression to castration‐resistant PCa (CRPC) in patients with advanced PCa. They emphasized the importance of identifying patients at high risk for rapid CRPC progression based on LUTS severity and other prognostic factors [85]. Recently, Koo et al., by focusing on primary care identification of malignancies, examined 1135 men who visited their general practitioner due to LUTS and were later diagnosed with PCa [86]. Within this specific group, they found that nearly 20% of diagnosed men already had Stage IV PCa with metastases. This percentage exceeds the national average rate of metastatic illness in men diagnosed by any method and is much greater than that of individuals assessed by elevated PSA levels. The exact origins of the causal connection between LUTS and cancer are difficult to trace, but they are believed to date back to the period before PSA testing was introduced, when formal research on this topic was limited [87].

Around a decade ago, the Gothenburg screening trial studied the occurrence of urinary symptoms and the identification of cancer in men with an elevated PSA level of ≥ 3 ng/mL. Contrary to expectations, the study did not discover any link; instead, it noted a negative correlation between symptoms and the likelihood of a positive biopsy [88]. Some studies have also shown no correlation between LUTS and PCa in men screened for suspected cancers [89, 90]. Age distribution could be a factor impacting the different results of these studies [90]. Additionally, some studies have shown a dichotomous distribution of findings on the prevalence of LUTS, only its presence or absence, without specifying the symptoms or degree of manifestation [82]. Therefore, males with LUTS should be examined for PCa. An Australian study indicated that 75% of patients with LUTS were likely to be tested for PCa, as recommended by the National Health and Medical Research Council [91]. Table 1 summarizes the recent studies that have investigated the relationship between UTIs and PCa. While some findings indicate a higher risk of PCa following recurrent UTIs, others highlight the role of urinary microbial dysbiosis and inflammation in cancer development, emphasizing the need for further research on diagnostic and therapeutic implications.

**Table 1:** Summary of studies on the relationship between UTIs and PCa.

Objective	Methods	Key findings	Implications	Ref.
Investigate if LUTIs are associated with an increased risk of PCa	Nationwide cohort study using the Taiwan Longitudinal Health Insurance Database	Cystitis and urethritis increase PCa risk. Risk is higher with frequent LUTI visits	Suggests LUTI, especially with repeated visits, may contribute to PCa development	[11]
Examine the relationship between PCa risk, antibiotics, and LUTIs	Matched case‐control study using Swedish PCBaSe 3.0 data	Short‐term UTIs or antibiotic prescriptions linked to increased PCa risk. No significant direct link between UTIs and PCa risk overall	Indicates potential detection bias; highlights the importance of diagnostic workup in PCa risk assessment	[92]
Investigate the link between UTI, antibiotic use, and cancer risk	Retrospective cohort study using insurance data (2009–2013); 38,084 UTI patients and 76,168 controls	UTI associated with increased cancer risk (aHR = 1.32 for males, 1.21 for females); higher risk for genitourinary cancers, especially in men	UTI may indicate early genitourinary cancers; careful antibiotic use during UTI treatment is advised	[93]
Determine prevalence, risk factors, and antimicrobial sensitivity for UTIs in BPH and PCa patients	Examination of individuals with PCa or BPH‐related bladder outlet obstruction	35.6% of patients had de novo UTIs, with *E. coli* being the predominant pathogen. Indwelling catheters are a key risk factor	Highlights the need for UTI prevention strategies and effective management in BPH and PCa patients	[94]
Investigate the role of urine flora in PCa and BPH	DNA sequencing of urine flora from PCa, BPH, and healthy controls; bioinformatic analysis	Microbial structure differed in PCa and BPH compared to controls; higher *E. coli* and lower *Lactobacillus* levels in PCa/BPH	Urinary microbial imbalance may serve as a biomarker for risk prediction and early treatment of prostate diseases	[95]
Explore the association between urinary microbiota and PCa pathogenesis	Retrospective study from NHIRD	PCa patients have a higher risk of UTIs. An abnormal urine microbiome may contribute to PCa through inflammation and immune dysregulation	Suggests a role for urine microbiome in PCa pathogenesis; potential for microbiome‐targeted therapies	[96]
Assess the impact of UTIs on prognosis in PCa patients postradiation therapy and radical prostatectomy	Population‐based study using the National Health Insurance Service database	UTI incidence is higher posttreatment, with varying rates between radiation and surgery. Robot‐assisted RP has a lower UTI risk compared to other methods	UTI management is critical for prognosis; treatment modality affects UTI risk	[97]
Analyze bacterial microbiota in urine, glans, and prostate biopsies of PCa vs. non‐PCa patients	16S rRNA sequencing of microbiota from urine, glans, and prostate	Dysbiosis was observed in the urine of PCa patients with higher levels of *Streptococcus*, *Prevotella*, and so forth. Lower microbiota diversity in PCa prostate	UTI‐associated bacteria in PCa may drive chronic inflammation and PCa progression	[98]
Investigate UTI as a risk factor for infection following prostate biopsy and its relation to PCa	Evaluation of urine culture routine before biopsy; identification of risk factors for infection	Urine culture before biopsy did not lower infection rates; previous UTIs were identified as a significant risk factor for postbiopsy infection in PCa patients	Emphasizes the need to consider UTI history in PCa patients undergoing biopsies to mitigate infection risks	[99]
Explore the role of urinary microbiota in PCa development and treatment	Comprehensive literature review	Urinary microbiota may influence PCa formation and treatment outcomes; it identifies microbial signatures linked to cancer	Potential for using urinary microbiota as biomarkers for diagnosis and patient stratification; implications for precision medicine	[100]
Explore UMB′s role in urological cancers, including PCa	Literature review using PubMed data	UMB changes linked to bladder cancer; PCa has more proinflammatory bacteria	Insights may lead to new therapies and risk classification	[101]

Managing UTIs in individuals with PCa can be more complex due to the potential impact of cancer treatment on the immune system and overall health of the individual [12]. In one study, the frequency of UTIs in patients with PCa who underwent RT or RP was significantly higher than that in the general population. Patients who underwent RP had a greater risk of UTIs than those who underwent RT during the initial follow‐up period [97]. Certain antibiotics commonly used to treat UTIs may interact with PCa medications or treatments, requiring careful consideration by healthcare providers [102]. For instance, broad‐spectrum antibiotics have a negative predictive impact on immune checkpoint inhibitor treatment outcomes in cancer patients, likely due to ATB‐induced dysbiosis [103, 104].

While LUTI alone did not impact PCa risk, having these infections or receiving antibiotics 6–12 months before the diagnosis of PCa increased the risk of PCa. Interestingly, PCa was less common in men who were prescribed at least 10 antibiotics, suggesting a complex relationship between infections, antibiotics, and PCa risk, which may be influenced by detection bias [92]. Early and accurate diagnosis of UTIs in individuals with PCa is crucial to prevent the potential spread of infection to the prostate gland and to minimize complications [105]. Healthcare providers may need to tailor the treatment of UTIs in individuals with PCa, considering the individual′s specific health condition, stage of cancer, ongoing treatments, and any potential interactions between antibiotics and cancer medications [102]. Reports indicate that statins, which are HMG‐CoA reductase inhibitors, can decrease the chances of developing UTI by lowering susceptibility to bacteria [106]. This evidence suggests a possible association between UTIs, LUTS, urinary microbial dysbiosis, and PCa; however, the findings remain inconsistent and are frequently confounded by age, screening practices, antibiotic use, and detection bias. Emerging data support a role for chronic inflammation and alterations in the urinary microbiome in creating a protumorigenic environment within the prostate. Further longitudinal and mechanistic studies are needed to clarify the contribution of UTIs to PCa development and progression.

### 4.4. Catheterization

Urethral catheterization is a frequently performed procedure for acute urinary retention (AUR) treatment [107]. Some studies have investigated the effects of urethral catheterization on PSA levels and the incidence of PCa [108–110]. AUR, as the biggest challenge in BPH, can lead to elevated PSA levels, which complicates the assessment of cancer through preoperative biopsies. Because PSA serves as a marker for PCa, many individuals with BPH and a catheter in place may undergo prostate biopsies unnecessarily to rule out cancer [111]. It has been suggested that these patients could safely proceed to surgery without biopsy‐related delays and that long‐term monitoring of postoperative PSA is a stronger predictor of cancer risk than preoperative PSA levels alone [109]. This approach would lead to more rapid cessation of catheter and avoid health complications of biopsy, such as rectal bleeding, complicated UTI, hematuria, and bloody semen, though at the expense of the percent whose acute episode is cured by temporary catheter.

This situation could lead to substantial health complications such as rectal bleeding, complicated UTI, hematuria, and bloody semen. Some studies have indicated a notable correlation between indwelling urethral catheters, prostatic inflammation, and increased serum PSA levels [112–114]. However, contrasting findings suggest that the presence of an indwelling urethral catheter does not lead to any significant clinical or statistical alterations in PSA levels [110, 115–117]. A study suggested that while AUR and catheterization may contribute to elevated PSA levels, prostate biopsies may not be needed for patients with a PSA level < 10 ng/mL and a prostate volume exceeding 60 mL [109]. Faris et al. reported that placing a catheter in BPH patients experiencing urinary retention led to a significant increase in serum PSA levels only in those who already had elevated PSA at baseline. However, in patients with normal baseline PSA levels, no significant changes were observed. These findings suggest that while catheterization can influence PSA levels, the effect depends on pre‐existing PSA levels. Therefore, careful evaluation is necessary to differentiate between PSA elevation due to catheterization and potential underlying malignancy [118]. UTI can cause an increase in PSA levels due to the proximity of the urinary tract to the prostate. Infection can inflame and irritate prostate cells, leading to an elevation in PSA concentration, which reflects the natural defense response of the prostate. One study showed that total PSA levels were significantly higher in patients with UTI than in those without UTI [119]. While PSA can potentially indicate PCa, current guidelines typically do not advise testing PSA for all patients with acute bacterial prostatitis [120]. Another study investigated the prevalence and predictors of UTI in patients with BPH and PCa. These findings showed that 35.6% of patients developed de novo infections, primarily driven by Gram‐negative bacteria such as *E. coli*. The only independent predictor of UTI was the existence of an indwelling catheter, emphasizing the importance of preventive measures against catheter‐associated infections [94].

In summary, catheterization in patients with AUR can elevate PSA levels, which complicates PCa assessment. However, this does not always indicate malignancy, and unnecessary biopsies should be avoided, especially in those with PSA < 10 ng/mL and prostate volume > 60 mL. UTIs, which are common in catheterized patients, further contribute to elevated PSA levels. A selective approach that prioritizes long‐term PSA monitoring over immediate biopsy may reduce complications and improve patient care.

### 4.5. Prostate Microbiome

In the past 20 years, several research efforts have been conducted to understand the microbiome and its impact on the development of PCa. Progress in analytical methods and contamination prevention has revealed a possible natural microbiome in prostate tissue, with microbial imbalance (dysbiosis), possibly contributing to PCa. The microbiome may contribute to inflammation and the development of LUTI, prostatitis/chronic pelvic pain syndrome, BPH, and PCa [121, 122].

Examination of the microbiome of both PCa and healthy tissues showed that there were higher levels of *Staphylococcus* spp. and *Propionibacterium* spp. present in the tumor and surrounding tissues [123]. Another study highlighted that *Propionibacterium acnes* (*P. acnes*), also known as *Cutibacterium acnes*, was consistently found as the predominant species and plays a significant role in promoting proinflammatory responses in prostate tissue in experiments involving mice [124]. Similar studies have investigated the association between circulating levels of *P. acnes* antibodies and PCa risk. These findings suggested that higher *P. acnes* antibody levels were associated with a reduced risk of PCa, especially in advanced disease stages [92, 125, 126]. Moreover, Sutcliffe et al. found that men who had used tetracycline for four or more years (to indicate a history of severe acne) had a notably increased risk of developing PCa. These results support the hypothesis that *P. acnes* may have a role in PCa [127]. *P. acnes* is widely recognized for its ability to induce cytokine production and inflammation. Long‐term NF‐*κ*B activation was induced by *P. acnes*, indicating significant reprogramming of the host cell′s inflammatory signaling. Prolonged contact with *P. acnes* alters cell division and triggers cellular changes. Furthermore, *P. acnes* has been linked to a robust, multidimensional inflammatory response with elevated IL‐6 levels, which is linked to advanced PCa metastases [12].

Early onset of puberty and a history of acne have been proposed as risk factors for PCa [128]. Long‐term follow‐up studies, including the Glasgow Alumni Cohort, demonstrated that men with a history of severe acne in youth experienced higher PCa‐specific mortality decades later [129], while another cohort linked late‐adolescent acne with increased PCa risk [130]. Severe acne is frequently associated with persistent colonization by *P. acnes*, a bacterium consistently detected as the predominant species in PCa [131, 132], strengthening the hypothesis that puberty‐acquired acne may serve as a marker of PCa susceptibility.

A recent metagenomics study comparing microorganisms in PCa tissue samples from African and European patients identified 23 bacterial genera in samples of tumors. The most prevalent strains in all samples were *Propionibacterium*, *Escherichia*, and *Pseudomonas*. The study observed increased bacterial richness in African‐derived tumor tissues, with notable enrichment of *Escherichia* and *Acidovorax*. Additionally, *Eubacterium* abundance was associated with tumor hypermutation in African patients. These findings suggest a potential bacterial contribution to oncogenic transformation, which may be relevant to the observed disparities in PCa aggressiveness [133]. Banerjee et al. identified a microbiome profile for PCa and hypothesized that *Helicobacter cagA* sequences incorporated into particular prostate tumor cell chromosomes would influence the expression of many oncogenic process‐related cellular genes [134]. Moreover, it has been indicated that specific bacterial signatures are linked to PCa in patients from India. While *Cupriavidus taiwanensis* and *Methylobacterium organophilum* were particularly high in PCa samples, the most prevalent bacteria in diseased prostate lesions were *Prevotella copri*, *Cupriavidus campinensis*, and *P. acnes*. Moreover, these findings suggest that microbial signatures could serve as potential biomarkers for the early diagnosis and prognosis of PCa [135].

It has been shown that PCa tissue had reduced microbial diversity compared to benign tissue, with significant decreases in *Staphylococcus saprophyticus* and *Vibrio parahaemolyticus* and an overrepresentation of *Shewanella*. Additionally, *Microbacterium* species were more abundant in advanced (T3) tumors than in early‐stage (T2) tumors. High *Shewanella* levels were associated with downregulated Toll‐like receptor (TLR) signaling and reduced dendritic cells, while low *V. parahaemolyticus* levels were correlated with the enrichment of olfactory transduction and drug metabolism pathways. Malignant samples also showed higher enrichment of M1 and M2 macrophages compared to benign tissues [136]. A study utilizing large‐scale RNA sequencing and clinical data from PCa patients found that certain intratumor microbes play an anticancer role by recruiting immune cells. The PCa Gleason score, tumor–node–metastasis (TNM) stage, AR expression, and PSA level all showed unfavorable correlations with these microorganisms. Specific bacteria, including *Listeria monocytogenes* and *Methylobacterium radiotolerans*, have been identified as potential contributors to reduced cancer aggressiveness [137].

**Figure 2:**
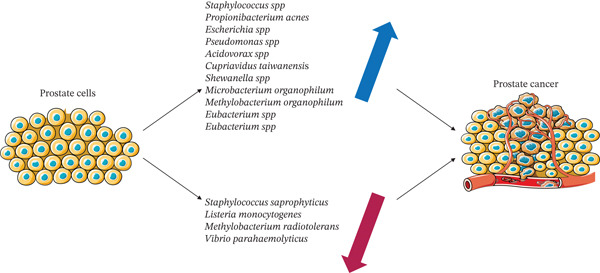
Influence of prostate microbiomes on cancer risk. An increase in some microbiomes (blue arrow) or a decrease in other microbiomes (red arrow) may increase the risk of PCa progression.

In conclusion, the prostate microbiome plays a key role in PCa progression and development. While certain bacterial species have been linked to chronic inflammation and tumor‐promoting effects, others may have antitumor properties by recruiting immune cells. Differences in microbial diversity between malignant and benign tissues suggest that bacterial signatures can serve as biomarkers for diagnosis and treatment. Further research is needed to explore their therapeutic potential in PCa. Figure 2 illustrates how alterations in the prostate microbiome can influence cancer risk, with increased or decreased microbial presence being linked to tumor progression.

### 4.6. Sexually Transmitted Infections (STIs)

Evidence points to a potential correlation between STIs and an elevated risk of developing PCa [138, 139]. It has been hypothesized that a past medical record of recurring STIs or unmanaged infections may significantly increase the development of PCa [140]. Initial suggestions linking STIs to the onset of PCa extend back to the 1950s [141]. Exogenous pathogens can trigger long‐lasting inflammation in prostate tissue, resulting in uncontrolled cell proliferation and potentially initiating the development of cancer [142].

For example, it has been shown that high‐risk human papillomaviruses (HPVs) are strongly linked to PCa, with studies detecting them in 22.6% of cancer cases versus 8.6% of benign tissues. HPVs may contribute to prostate oncogenesis through chronic inflammation, cellular immortalization, and interactions with APOBEC enzymes and other pathogens [143]. Moreover, a recent review article conducted by Lawson and Glenn demonstrated that PCa had a significantly greater prevalence of high‐risk HPV DNA than normal and benign prostatic controls in 10 out of 27 case‐control studies using PCR methods [140]. High‐risk HPVs have been demonstrated to exist in benign prostate tissues before the emergence of HPV‐positive PCa, with benign tissues exhibiting noticeably greater HPV E7 oncoprotein expression than later PCa [144]. Moreover, in a study comparing the oncogenic potential of high‐risk HPVs in human foreskin keratinocytes, it was found that HPV16, ‐31, ‐33, and ‐35 strongly induced immortalization, while others did so less frequently or after the crisis phase. In contrast, HPV52 extended its lifespan but did not immortalize cells, highlighting variations in HPV‐driven cancer risk [145]. Importantly, in the context of transmission, men can serve as reservoirs of high‐risk HPVs, which contribute to the spread and persistence of the virus, thereby increasing the risk of cervical cancer [146]. In women, the presence of anaerobic bacteria is associated with bacterial vaginosis and ultimately increases the risk of persistent HPV infection and progression to cervical cancer [147]. Similarly, the prostate microbiome is enriched with anaerobic bacteria, which have been associated with PCa progression and poorer prognosis, suggesting they may interact with high‐risk HPVs and contribute to oncogenesis [148].

Another well‐known cause of STIs, gonorrhea, is *Neisseria gonorrhoeae* [149]. A study involving a large population of men in the United States found that those with a previous history of gonorrhea or syphilis had a higher risk of developing PCa [14]. This bacterium evades protective immunity through multiple mechanisms, including antigenic variation, blocking of antibodies, and induction of a less effective Th17 immune response. Human studies have shown limited immune memory, which prevents the development of protective immunity and allows repeated infections [150]. According to a meta‐analysis based on data from over 10,000 PCa cases in 23 different studies, males who had previously had a gonorrhea infection are 20% more likely to develop PCa [138]. Moreover, another meta‐analysis of 21 studies, including 9965 patients with PCa and 118,765 participants, found that gonorrhea was significantly associated with an increased risk of PCa. The association was stronger in African American males than in Whites [151].

Herpes simplex virus Type 2 (HSV‐2) is an STI that causes genital herpes [152]. Transmission occurs through sexual contact, and its symptoms include painful blisters or ulcers that may recur over time. However, the majority of HSV infections are asymptomatic or undetectable [153, 154]. Several studies have reported that the prevalence of HSV‐2 is higher in individuals with PCa [155–157]. Interestingly, a study of 534 US military personnel found no link between recent STIs and PCa but identified a significant association with prior HSV‐2 infection, especially in samples taken 60+ months before diagnosis, suggesting a possible long latency period [156].

STDs caused by bacteria, such as *Chlamydia trachomatis* and *Mycoplasma genitalium*, have been explored as potential factors contributing to the development of PCa [158, 159]. Nevertheless, no positive correlations between *C. trachomatis* infections and PCa have been found in many case‐control studies. However, since all these studies rely on serology, these case‐control studies are likely misleading because *C. trachomatis* may be the cause of persistent prostate infections that result in PCa. In both benign prostate controls and PCa, this results in positive antibodies [140, 160, 161]. On the other hand, in three analyses based on PCR, it has been found a significant association between *Mycoplasma hominis* and PCa [162–164].

The most common nonviral STD in the world, trichomoniasis, is caused by *Trichomonas vaginalis* [165]. Men who have an infection with *T. vaginalis* may develop prostatitis, urethritis, decreased fertility, and an increased risk of contracting the human immunodeficiency virus. Furthermore, men can unintentionally infect their female sexual partners with the virus because they are frequently asymptomatic [166]. A study found that *T. vaginalis* seropositivity was significantly higher in PCa patients (19%) than in healthy controls (8.3%), suggesting a potential association. However, no significant correlation was found between *T. vaginalis* infection and overall survival or other prognostic factors, indicating that while infection may be linked to PCa, it does not appear to worsen the disease [167]. Kim et al. found that men with prostatic tumors, including BPH and PCa, had a significantly higher seropositivity to *T. vaginalis* (19.7%) than healthy controls (1.7%). Specifically, seropositivity was 18.7% in patients with BPH and 22.7% in patients with PCa. This suggests a potential association with *T. vaginalis* [168]. However, Tsang et al. conducted a large serology‐based study, and it has been shown that there is no association between *T. vaginalis* serostatus and PCa‐specific or all‐cause mortality in two large cohorts (PHS and HPFS), despite prior evidence suggesting a link to PCa development [169].

Inflammatory conditions triggered by sexually transmitted microorganisms have the potential to influence the innate immune responses in the body. This influence becomes more noticeable when specific genetic variations and associated proteins are present [170]. TLRs, a type of pattern recognition receptor (PRR), are among the genes that could play a role in this situation, as some of their variants have been linked to either an elevated or decreased risk of PCa. TLRs activate genes that produce inflammatory cytokines and chemokines that help eliminate microorganisms [171]. However, more research is required because there is currently an absence of precise prospective evidence about the precise processes of STIs in the development of PCa.

Overall, epidemiological and mechanistic evidence suggests that certain STIs may contribute to prostate carcinogenesis primarily through persistent inflammation and immune dysregulation. Among these, high‐risk HPV and gonorrhea show the most consistent associations with increased PCa risk, while HSV‐2 may involve a prolonged latency period. Evidence for other pathogens, such as *C. trachomatis*, *M. hominis*, and *T. vaginalis*, remains less consistent, often reflecting variations between serology and PCR detection methods across study populations. Collectively, these observations support a possible role of STI‐driven chronic infection and host immune responses in PCa development.

## 5. Inflammatory Signaling Pathways in PCa and Therapeutic Targets

Numerous phases of tumor growth, such as initiation, promotion, invasion, malignant conversion, and metastasis, are significantly influenced by inflammatory responses. Additionally, inflammation influences immunological surveillance and treatment responses [172]. While acute inflammation protects against detrimental stimuli and initiates tissue repair, chronic inflammation creates a protumorigenic environment characterized by the persistent production of inflammatory mediators such as chemokines, cytokines, and reactive oxygen species (ROS) [172]. Persistent inflammatory stimuli, often arising from infection, tissue injury, or chronic irritation, create a microenvironment rich in cytokines, chemokines, and ROS that promote DNA damage, impair repair mechanisms, and result in permanent genomic alterations. These processes facilitate the accumulation of oncogenic mutations and support the survival and proliferation of transformed cells. In this context, inflammation functions as a “tumor‐promoting” condition by enabling proliferative signaling in cells that have lost normal growth control [173, 174].

The risk of chronic prostate inflammation is increased by several factors, including bacterial infections, viruses, age, diet, hormones, environmental exposure, autoimmune reactions, and some drugs. These elements cause genetic and epigenetic changes over time [175, 176].

Beyond tumor initiation, inflammatory cells actively shape the tumor microenvironment and directly contribute to cancer progression and metastasis. Tumor‐associated immune cells, particularly macrophages, neutrophils, and mast cells, secrete growth factors such as VEGF, TGF‐*β*, and PDGF, as well as proteases and angiogenic mediators that promote extracellular matrix remodeling, angiogenesis, and lymphangiogenesis. These processes not only enhance tumor growth and invasion but also facilitate the dissemination of malignant cells to distant organs. Furthermore, cancer cells exploit inflammatory signaling pathways, including chemokines and adhesion molecules, to promote metastatic homing [173, 177]. Collectively, these interactions establish inflammation as a key functional component of cancer biology and a critical therapeutic target in oncology. The progression and development of PCa are fueled by these alterations, which deactivate tumor suppressor genes and activate oncogenes [176]. Chronic inflammation drives PCa progression by activating various signaling pathways [178]. The following sections intended to summarize the major inflammatory mechanisms implicated in prostate carcinogenesis. These sections explore key inflammatory signaling cascades, including NF‐*κ*B, signal transducer and activator of transcription 3 (STAT3), and cyclooxygenase‐2/prostaglandin E_2_ (COX‐2/PGE_2_), highlighting their mechanistic contributions to PCa and their potential therapeutic targets.

### 5.1. NF‐*κ*B Pathway

In PCa, activation of the NF‐*κ*B pathway sustains AR activity, promoting androgen‐independent (AI) PCa progression. In LNCaP (lymph node carcinoma of the prostate) cells, secretory proteins from neuroendocrine cells stimulate NF‐*κ*B, which raises AR levels; in contrast, inhibiting NF‐*κ*B signaling prevents AR activation. Continuous NF‐*κ*B activation prevents prostate regression after castration in vivo by maintaining nuclear AR and epithelial proliferation [179]. It has been shown that nuclear localization of NF‐*κ*B/p65 is associated with PCa progression and biochemical relapse. Nuclear NF‐*κ*B, but not cytoplasmic NF‐*κ*B, was significantly linked to biochemical relapse, and multivariate analysis confirmed it as an independent prognostic factor alongside PSA levels and Gleason score [180]. Moreover, chemoresistance and radioresistance are caused by the overexpression of NF‐*κ*B, and antitumor drugs that inhibit NF‐*κ*B can slow the progression of cancer [181]. IKK*β* or constitutive IKK complex‐independent activation of NF‐*κ*B stimulates the development of CRPC [182].

A platelet‐activating factor (PAF) antagonist called PMS1077 targets IKK‐*β* to prevent TNF‐*α* from activating the NF‐*κ*B signaling pathway. PMS1077 suppressed I*κ*B‐*α* phosphorylation and degradation, blocked p65 phosphorylation and nuclear translocation, and downregulated NF‐*κ*B‐regulated antiapoptotic genes, thereby sensitizing cells to TNF‐*α*‐induced apoptosis [183]. Bortezomib has been demonstrated to prevent the activation of NF‐*κ*B by preventing the breakdown of I*κ*B by proteasomes in different cancers, including PCa [184]. Moreover, curcumin, a naturally occurring NF‐*κ*B inhibitor that targets the NF‐*κ*B pathway, is a promising strategy for treating PCa by interfering with important processes that contribute to tumor growth and treatment resistance [185].

### 5.2. The STAT3

This pathway plays a significant role in PCa progression [186]. It has been found that combining enzalutamide with the STAT3 inhibitor GPB730 significantly enhanced growth inhibition in PCa cells and reduced the IC50 of enzalutamide. GPB730 reduced the expression of PSA and amplified enzalutamide‐induced suppression of NKX3.1, c‐Myc, and survivin, which are key regulators of cancer progression [187]. Several signaling pathways implicated in the development of PCa to metastatic illness have been influenced by STAT3. When ligands attach to either receptor tyrosine kinases or cytokine receptors, nonreceptor tyrosine kinases (such as Src and Janus tyrosine family kinase [JAK] families) are attracted to the receptors via their SH2 domains. Consequently, nonreceptor kinases phosphorylate STAT3 on Tyrosine 705, which causes its dimerization, nuclear translocation, and attachment to the DNA of target genes related to angiogenesis (Hif1*α* and VEGF), proliferation (CyclinD1, Mcl1, and cMyc), and EMT (MMP‐2, 9, 7, and Twist). Furthermore, STAT3 is phosphorylated at Serine 727 by the mTOR and mitogen‐activated protein kinase (MAPK) pathways, which, without increasing cell proliferation, directly interact with the AR′s NTD to promote its differentiation action [188].

Moreover, adipocytes have been shown to contain the JAK1/2 and ruxolitinib small‐molecule inhibitors. STAT3 can be rendered inactive by indirectly interfering with its phosphorylation through the inhibition of JAK2 proteins [189]. Additionally, resveratrol, a naturally occurring phytoalexin, inhibited the STAT3 signaling pathway to cause apoptosis in human PCa cells [190]. Furthermore, the flavanone silibinin, which is derived from silybum, can prevent constitutive activation of STAT3 and trigger activation of caspase and human PCa cell apoptosis [191].

### 5.3. The COX‐2/PGE_2_ Pathway

PGE2 is the most prevalent proinflammatory mediator in prostate tissue, and PCa has been linked to elevated PGE2 levels. PGE2 has several functions, such as boosting antiapoptotic proteins, controlling the immune system, and encouraging the development, proliferation, invasion, and metastasis of cancer cells [192–194]. Additionally, in PCa, COX‐2 has a favorable correlation with tumor lymphangiogenesis, VEGF‐C expression, and lymphatic metastasis [195]. Through the cAMP PKA/PI3K Akt signaling pathway, PGE2 markedly increased the mRNA and protein expression levels of MMP‐2, MMP‐9, RUNX2, and RANKL. Additionally, the EP4 receptor contributed to PCa cell invasion and proliferation [194].

It has been demonstrated that celecoxib, a specific Cox‐2 inhibitor, effectively reduces prostate tumor development and tumor angiogenesis [196]. A 55% reduction in PCa risk associated with selective COX‐2 inhibitor use has been reported in patients with autoimmune conditions, such as rheumatoid arthritis, who received these drugs for more than 2 years [197]. Furthermore, by inhibiting COX, anti‐inflammatory medications such as nonsteroidal anti‐inflammatory drugs (NSAIDs) can interfere with the tumor microenvironment by decreasing cell migration and enhancing chemosensitivity and apoptosis [198].

### 5.4. The IL‐6 and IL‐8 Signaling Pathways

Patients with untreated metastatic PCa or CRPC had higher serum IL‐6 levels, which were inversely linked to tumor survival and treatment response. IL‐6 can stimulate PCa cell proliferation and prevent apoptosis in vitro and in vivo through a variety of signaling pathways, such as the JAK‐signal transducer and activator of transcription (STAT) pathway, phosphoinositide 3‐kinase (PI3‐K) pathway, and extracellular signal–regulated kinase 1 and 2 (ERK1/2)–MAPK pathway. It is associated with an aggressive PCa phenotype and may contribute to the process of metastasis by regulating the EMT and bone homing of cancer cells [199, 200].

PCa and other malignancies have been found to have elevated levels of IL‐8, a crucial proinflammatory chemokine that interacts via the G protein‐coupled receptors (GPCRs) CXCR1 and CXCR2. IL‐8 plays a crucial role in inflammation and tumor development, promoting the migration, survival, and resistance of cancer cells to treatment [201]. Aggression and AR loss in primary and metastatic PCa are linked to IL8 expression [202]. Additionally, by focusing on the IL‐8 receptor, CXCR2, researchers have tried to create novel medications that will treat individuals with resistant forms of PCa and increase the efficacy of current therapies [201]. Siltuximab is an anti‐interleukin‐6 chimeric monoclonal antibody. However, in a Phase II trial evaluating mitoxantrone/prednisone with or without siltuximab in patients with metastatic CRPC, there have been found that no improvement in progression‐free survival (PFS) was found, and it was associated with a higher frequency of abnormal laboratory findings [203]. BMS‐986253 is a human monoclonal antibody that inhibits IL‐8. In a Phase I trial of examining this drug in patients with metastatic or unresectable solid tumors, it has been shown that this is safe and well‐tolerated in patients with advanced solid tumors, with no dose‐limiting toxicities and mostly mild side effects. Although no objective tumor responses were observed, 73% of patients achieved stable disease [204].

### 5.5. The TLR Pathway

Both healthy and cancerous prostate epithelial cells express TLR4 [205]. The TLR4 signaling pathway allows cells to respond to bacterial infections by sensing lipopolysaccharide (LPS) on bacterial cell walls [206]. In response to invasive infections, innate immune responses are triggered when prostate cells activate TLR4 signaling. However, tumor cell activation, growth, survival, and transformation can be increased by persistent stimulation of the TLR4 signaling pathway in prostate epithelial cells [205, 207]. Components of bacterial and viral pathogens, represented experimentally by LPS and CpG DNA as surrogates for Gram‐negative bacteria and DNA viruses, may contribute to the malignant transformation of benign prostate epithelial cells via TLR4‐ and TLR9‐dependent signaling pathways. These pathways lead to increased proliferation, enhanced resistance to apoptosis, and activation of NF‐*κ*B signaling [207]. In PCa, TLR4 expression and the resulting chronic inflammation are associated with poor treatment outcomes and accelerated disease progression [208]. Furthermore, through elevated TLR4 expression and generation of proinflammatory mediators, prostate epithelial cells upregulate NF‐*κ*B, TGF‐*β*1, and VEGF [208, 209]. TLR4 plays a crucial role in PCa metastasis, with higher expression levels correlating with increased invasiveness of PCa cell lines. Gene silencing of TLR4 using siRNA significantly suppressed TLR4 expression and reduced the migration, invasion, and viability of highly metastatic PC‐3 cells while inducing apoptosis. Inhibition of TLR4‐mediated signaling disrupted key downstream molecules, including MyD88, TRIF, and IRF‐1. In a mouse model, siRNA targeting of TLR4 effectively suppressed tumor growth and improved survival [210]. The most prevalent abnormality of the PCa genome is the TMPRSS2‐ERG gene fusion, which leads to overexpression of the ERG transcription factor in around half of PCa cases. The TLR4 signaling pathway is a crucial regulator of ERG function in ERG‐positive PCa. While ERG is known to activate TLR4 gene expression, the study reveals that TLR4 signaling also enhances ERG phosphorylation at Serine 96, promoting its transcriptional activity. Inhibition of TLR4 effectively reduces ERG‐mediated tumorigenic properties, including migration, clonogenic survival, and tumor growth [206]. Activation of TLR4 by bacterial LPS improves PCa cell survival in nutrient‐stressed environments through mechanisms involving CCL2 upregulation and suppression of starvation‐induced macroautophagy, suggesting that bacterial infection and TLR4 activation may contribute to PCa pathogenesis [211]. TLR9 is expressed in PCa cells, and its activation by CpG‐oligonucleotides or bacterial DNA enhances cancer cell invasion via matrix metalloproteinase‐13 (MMP‐13). This effect was only observed in TLR9‐positive cells and was blocked by chloroquine or anti‐MMP‐13 antibodies, supporting the hypothesis that infection‐driven TLR9 activation may represent a novel mechanism by which infections promote PCa invasion and progression [212].

### 5.6. The NLRP3 Inflammasome Pathway

It was demonstrated that, compared to the healthy cell line, the expression level of NLRP3 was significantly higher (at both the mRNA and protein levels) in PCa cell lines. However, heterogeneous immunostaining for NLRP3 has been observed in human PCa tissues compared with normal surrounding tissue [213]. PCa tissues and cell lines have elevated levels of the NLRP3 inflammasome, which activates caspase‐1 to promote malignant development. In PCa cells, activation of the NLRP3/caspase‐1 inflammasome by LPS + ATP promoted migration and proliferation, while preventing apoptosis. In vitro and in vivo, NLRP3 overexpression enhanced tumor aggressiveness, whereas its knockdown inhibited tumor growth [214]. Additionally, PCa cells may express more programmed death‐ligand 1 (PD‐L1) due to the NLRP3 inflammasome, which suppresses the CD8+ T cell‐mediated antitumor immunological response [215, 216]. Additionally, Panchanathan et al. showed that hypoxia stabilizes NLRP3 posttranscriptionally. Hypoxia primed normal human prostate cells, benign BPH‐1, and tumor PC‐3 cell lines and increased NLRP3 inflammasome activation [217]. Studies have shown that inhibiting the NLRP3 pathway in PC‐3 cells and PCa‐derived xenografts significantly impacts the growth of tumors by interfering with cell migration, proliferation, and/or activating apoptosis [218]. MCC950, an NLRP3 inhibitor, decreased the expression of NLRP3, ASC, and caspase‐1 in a model of experimentally induced autoimmune prostatitis in mice and enhanced chronic pain test results [219].

In conclusion, chronic inflammation plays a pivotal role in PCa initiation and progression by activating key signaling pathways such as NF‐*κ*B, STAT3, COX‐2/PGE_2_, IL‐6/IL‐8, TLR, and NLRP3. These pathways drive oncogenic transformation, tumor growth, metastasis, and immune evasion. Targeting inflammation‐related mechanisms with small‐molecule inhibitors and natural compounds offers promising therapeutic strategies for PCa management. Further research is essential to optimize these approaches and identify novel anti‐inflammatory targets to improve patient outcomes.

**Figure 3:**
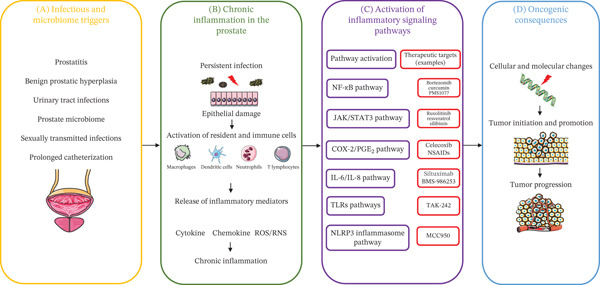
Proposed model illustrating the role of infection‐ and microbiome‐associated chronic inflammation in PCa. Infectious and microbial factors initiate persistent inflammatory responses within the prostate, leading to epithelial damage, activation of innate and adaptive immune cells, and sustained release of proinflammatory mediators. Chronic activation of NF‐*κ*B, JAK/STAT3, COX‐2/PGE_2_, IL‐6/IL‐8, TLR, and NLRP3 inflammasome signaling pathways creates a protumorigenic microenvironment that drives genomic and molecular alterations, ultimately facilitating tumor initiation, promotion, and progression.

Figure 3 provides a proposed link between infectious/microbiome‐associated prostate inflammation and prostate carcinogenesis.

## 6. Conclusion

Reviews of the studies suggest a biological link between chronic infections, persistent inflammation, and PCa development; however, the strength and consistency of this association vary considerably across different infectious agents and study designs. While prostatitis, certain STIs (particularly high‐risk HPV and gonorrhea), and microbial dysbiosis show more consistent associations with PCa risk, other conditions, such as BPH‐related inflammation, UTIs, and several bacterial pathogens, demonstrate conflicting or weak evidence, often influenced by detection and selection biases. Importantly, most current findings are derived from observational studies, limiting causal inference and making it difficult to distinguish true etiological roles. Therefore, infections should be considered potential modulators of PCa rather than established causal factors.

This review has some limitations that should be acknowledged. First, evidence is derived from a wide range of observational, experimental, and microbiome studies, which restricts the ability to infer causality between infections and PCa development. Second, heterogeneity in study design, patient populations, diagnostic methods, and microbial detection techniques complicates direct comparisons across studies. Moreover, one of the biggest challenges in studying the link between infections and PCa is detection bias. This happens when individuals diagnosed with infections are more likely to undergo PSA screening and biopsies, which can lead to an overdiagnosis of PCa. Future research should focus on large‐scale prospective studies to establish a causal relationship between infections and PCa rather than relying solely on retrospective or cross‐sectional analyses. These studies should apply advanced molecular profiling techniques, such as microbiome analysis and immune system characterization, to elucidate the specific microbial and inflammatory signatures associated with PCa. In this way, future epidemiological studies need to account for detection bias by adjusting for confounding factors. Using standardized diagnostic criteria and strong statistical models will help distinguish genuine associations from chance findings. Furthermore, treatments that focus on reducing inflammation caused by infections should be explored as possible strategies for preventing and treating PCa. Researchers should further explore whether anti‐inflammatory drugs, antibiotics, probiotics, and treatments that balance the microbiome can help lower the risk of PCa or slow its progression. Clinical trials are necessary to better understand their effectiveness and how they might be utilized in PCa prevention and treatment strategies.

**Nomenclature**
PCa: prostate cancer
BPH: benign prostatic hyperplasia
UTIs: urinary tract infections
LUTIs: lower urinary tract infections
STIs: sexually transmitted infections
STDs: sexually transmitted diseases
NF‐*κ*B: nuclear factor kappa‐light‐chain‐enhancer of activated B cells
STAT3: signal transducer and activator of transcription 3
COX‐2: cyclooxygenase‐2
PGE_2_
: prostaglandin E_2_
IL‐6: interleukin‐6
IL‐8: interleukin‐8
TLR: Toll‐like receptor
NLRP3: NOD‐like receptor family pyrin domain‐containing 3
CP/CPPSs: chronic prostatitis/chronic pelvic pain syndromes
CMHS: California Men′s Health Study
TURP: transurethral resection of the prostate
aHR: adjusted hazard ratio
NHIRD: National Health Insurance Research Database
UMB: urinary microbiota
BRCA: breast cancer genes
LNCaP: lymph node carcinoma of the prostate

## Author Contributions

**Ensiyeh Bahadoran:** writing—original draft, review, and editing, investigation, and visualization. **Abouzar Babaei, Shahla Shahbazi, Milad Badri, and Farhad Nikkhahi:** writing—review and editing. **Samira Sabzi:** writing—review and editing, visualization, and supervisor.

## Funding

This research received no external funding.

## Ethics Statement

This project was approved by the Ethics Committee of Qazvin University of Medical Sciences (IR.QUMS.REC.1403.473)

## Consent

The authors have nothing to report.

## Conflicts of Interest

The authors declare no conflicts of interest.

## Data Availability

No new data were created or analyzed in this review.

## References

[1] Bray F. , Laversanne M. , Sung H. , Ferlay J. , Siegel R. L. , Soerjomataram I. , and Jemal A. , Global Cancer Statistics 2022: GLOBOCAN Estimates of Incidence and Mortality Worldwide for 36 Cancers in 185 Countries, CA: A Cancer Journal For Clinicians. (2024) 74, no. 3, 229–263, 10.3322/caac.21834, 38572751. 38572751

[2] Bergengren O. , Pekala K. R. , Matsoukas K. , Fainberg J. , Mungovan S. F. , Bratt O. , Bray F. , Brawley O. , Luckenbaugh A. N. , Mucci L. , Morgan T. M. , and Carlsson S. V. , 2022 Update on Prostate Cancer Epidemiology and Risk Factors—A Systematic Review, European Urology. (2023) 84, no. 2, 191–206, 10.1016/j.eururo.2023.04.021, 37202314. 37202314 PMC10851915

[3] Guo Y. , Dong X. , Mao S. , Yang F. , Wang R. , Ma W. , Liu J. , Li C. , Zheng Z. , Zhang W. , Zhang A. , and Yao X. , Causes of Death After Prostate Cancer Diagnosis: A Population-Based Study, Oxidative Medicine and Cellular Longevity. (2022) 2022, no. 1, 8145173, 10.1155/2022/8145173, 35502209. 35502209 PMC9056212

[4] Kucera R. , Pecen L. , Topolcan O. , Dahal A. R. , Costigliola V. , Giordano F. A. , and Golubnitschaja O. , Prostate Cancer Management: Long-Term Beliefs, Epidemic Developments in the Early Twenty-First Century and 3PM Dimensional Solutions, EPMA Journal. (2020) 11, no. 3, 399–418, 10.1007/s13167-020-00214-1, 32843909. 32843909 PMC7429585

[5] Wang L. , Lu B. , He M. , Wang Y. , Wang Z. , and Du L. , Prostate Cancer Incidence and Mortality: Global Status and Temporal Trends in 89 Countries From 2000 to 2019, Frontiers in Public Health. (2022) 10, 811044, 10.3389/fpubh.2022.811044, 35252092. 35252092 PMC8888523

[6] James N. D. , Tannock I. , N′Dow J. , Feng F. , Gillessen S. , Ali S. A. , Trujillo B. , al-Lazikani B. , Attard G. , Bray F. , Compérat E. , Eeles R. , Fatiregun O. , Grist E. , Halabi S. , Haran Á. , Herchenhorn D. , Hofman M. S. , Jalloh M. , Loeb S. , MacNair A. , Mahal B. , Mendes L. , Moghul M. , Moore C. , Morgans A. , Morris M. , Murphy D. , Murthy V. , Nguyen P. L. , Padhani A. , Parker C. , Rush H. , Sculpher M. , Soule H. , Sydes M. R. , Tilki D. , Tunariu N. , Villanti P. , and Xie L. P. , The Lancet Commission on Prostate Cancer: Planning for the Surge in Cases, Lancet. (2024) 403, no. 10437, 1683–1722, 10.1016/S0140-6736(24)00651-2, 38583453. 38583453 PMC7617369

[7] Gnanapragasam V. J. , Greenberg D. , and Burnet N. , Urinary Symptoms and Prostate Cancer—The Misconception That May Be Preventing Earlier Presentation and Better Survival Outcomes, BMC Medicine. (2022) 20, no. 1, 10.1186/s12916-022-02453-7, 35922801. PMC9351095 35922801

[8] Kim M. S. and Jung S. I. , The Urinary Tract Microbiome in Male Genitourinary Diseases: Focusing on Benign Prostate Hyperplasia and Lower Urinary Tract Symptoms, International Neurourology Journal. (2021) 25, no. 1, 3–11, 10.5213/inj.2040174.087, 33504133. 33504133 PMC8022174

[9] Verze P. , Cai T. , and Lorenzetti S. , The Role of the Prostate in Male Fertility, Health and Disease, Nature Reviews Urology. (2016) 13, no. 7, 379–386, 10.1038/nrurol.2016.89, 27245504. 27245504

[10] Shahbazi S. , Karam M. R. A. , Habibi M. , Talebi A. , and Bouzari S. , Distribution of Extended-Spectrum *β*-Lactam, Quinolone and Carbapenem Resistance Genes, and Genetic Diversity Among Uropathogenic Escherichia coli Isolates in Tehran, Iran, Journal of Global Antimicrobial Resistance. (2018) 14, 118–125, 10.1016/j.jgar.2018.03.006, 29581075. 29581075

[11] Fan C. Y. , Huang W. Y. , Lin K. T. , Lin C. S. , Chao H. L. , Yang J. F. , Lin C. L. , and Kao C. H. , Lower Urinary Tract Infection and Subsequent Risk of Prostate Cancer: A Nationwide Population-Based Cohort Study, PLoS One. (2017) 12, no. 1, e0168254, 10.1371/journal.pone.0168254, 28046120. 28046120 PMC5207623

[12] Kustrimovic N. , Bombelli R. , Baci D. , and Mortara L. , Microbiome and Prostate Cancer: A Novel Target for Prevention and Treatment, International Journal of Molecular Sciences. (2023) 24, no. 2, 10.3390/ijms24021511, 36675055. PMC9860633 36675055

[13] Yu S. H. and Jung S. I. , The Potential Role of Urinary Microbiome in Benign Prostate Hyperplasia/Lower Urinary Tract Symptoms, Diagnostics. (2022) 12, no. 8, 10.3390/diagnostics12081862, 36010213. PMC9406308 36010213

[14] Hayes R. , Pottern L. , Strickler H. , Rabkin C. , Pope V. , Swanson G. , Greenberg R. S. , Schoenberg J. B. , Liff J. , Schwartz A. G. , Hoover R. N. , and Fraumeni J. F. , Sexual Behaviour, STDs and Risks for Prostate Cancer, British Journal of Cancer. (2000) 82, no. 3, 718–725, 10.1054/bjoc.1999.0986, 10682688. 10682688 PMC2363322

[15] Paulis G. , Inflammatory Mechanisms and Oxidative Stress in Prostatitis: The Possible Role of Antioxidant Therapy, Research and Reports in Urology. (2018) 10, 75–87, 10.2147/RRU.S170400, 30271757. 30271757 PMC6149977

[16] Bostanci Y. , Kazzazi A. , Momtahen S. , Laze J. , and Djavan B. , Correlation Between Benign Prostatic Hyperplasia and Inflammation, Current Opinion in Urology. (2013) 23, no. 1, 5–10, 10.1097/MOU.0b013e32835abd4a. 23159991

[17] Ebrahimzadeh T. , Basu U. , Lutz K. C. , Gadhvi J. , Komarovsky J. V. , Li Q. , Zimmern P. E. , and de Nisco N. J. , Inflammatory Markers for Improved Recurrent UTI Diagnosis in Postmenopausal Women, Life Science Alliance. (2024) 7, no. 4, e202302323, 10.26508/lsa.202302323, 38331474. 38331474 PMC10853434

[18] Galvin S. R. and Cohen M. S. , The Role of Sexually Transmitted Diseases in HIV Transmission, Nature Reviews Microbiology. (2004) 2, no. 1, 33–42, 10.1038/nrmicro794. 15035007

[19] Singh N. , Baby D. , Rajguru J. P. , Patil P. B. , Thakkannavar S. S. , and Pujari V. B. , Inflammation and Cancer, Annals of African Medicine.(2019) 18, no. 3, 121–126, 10.4103/aam.aam_56_18, 31417011. 31417011 PMC6704802

[20] Gonda T. A. , Tu S. , and Wang T. C. , Chronic Inflammation, the Tumor Microenvironment and Carcinogenesis, Cell Cycle. (2009) 8, no. 13, 2005–2013, 10.4161/cc.8.13.8985. 19550141

[21] Obukohwo O. M. , Kingsley N. E. , Rume R. A. , and Victor E. , The Concept of Male Reproductive Anatomy, Male Reproductive Anatomy, 2021, IntechOpen, 10.5772/intechopen.79167.

[22] Tang D. G. , Understanding and Targeting Prostate Cancer Cell Heterogeneity and Plasticity, Seminars in Cancer Biology. (2022) 82, 68–93, 10.1016/j.semcancer.2021.11.008. 34844845 PMC9106849

[23] Mangangcha I. R. , Malik M. Z. , Küçük Ö. , Ali S. , and Singh R. B. , Identification of Key Regulators in Prostate Cancer From Gene Expression Datasets of Patients, Scientific Reports. (2019) 9, no. 1, 16420, 10.1038/s41598-019-52896-x, 31712650. 31712650 PMC6848149

[24] Daryanani A. and Turkbey B. , Recent Advancements in CT and MR Imaging of Prostate Cancer, Seminars in Nuclear Medicine. (2022) 52, no. 3, 365–373, 10.1053/j.semnuclmed.2021.11.013. 34930627 PMC9038642

[25] Seibert T. M. , Garraway I. P. , Plym A. , Mahal B. A. , Giri V. , Jacobs M. F. , Cheng H. H. , Loeb S. , Helfand B. T. , Eeles R. A. , and Morgan T. M. , Genetic Risk Prediction for Prostate Cancer: Implications for Early Detection and Prevention, European Urology. (2023) 83, no. 3, 241–248, 10.1016/j.eururo.2022.12.021, 36609003. 36609003

[26] Bhanji Y. , Isaacs W. B. , Xu J. , and Cooney K. A. , Prostate Cancer Predisposition, Urologic Clinics of North America. (2021) 48, no. 3, 283–296, 10.1016/j.ucl.2021.03.001. 34210485

[27] Chen Y. , Lin P.-H. , Freedland S. J. , and Chi J.-T. , Metabolic Response to Androgen Deprivation Therapy of Prostate Cancer, Cancers. (2024) 16, no. 11, 10.3390/cancers16111991, 38893112. PMC11171316 38893112

[28] Ren R. , Koti M. , Hamilton T. , Graham C. H. , Nayak J. G. , Singh J. , Drachenberg D. E. , and Siemens D. R. , A Primer on Tumour Immunology and Prostate Cancer Immunotherapy, Canadian Urological Association Journal. (2016) 10, no. 1-2, 60–65, 10.5489/cuaj.3418, 26977209. 26977209 PMC4771561

[29] Taverna G. , Seveso M. , Giusti G. , Hurle R. , Graziotti P. , Stifter S. , Chiriva-Internati M. , and Grizzi F. , Senescent Remodeling of the Innate and Adaptive Immune System in the Elderly Men With Prostate Cancer, Current Gerontology and Geriatrics Research. (2014) 2014, 478126, 10.1155/2014/478126, 24772169. 24772169 PMC3977481

[30] Quallich S. A. , Quentin Clemens J. , Ronstrom C. , James A. S. , Kreder K. J. , Henry Lai H. , Naliboff B. D. , Rodriguez L. V. , Berry S. H. , and Sutcliffe S. , Flares and Their Impact Among Male Urologic Chronic Pelvic Pain Syndrome Patients: An In-Depth Qualitative Analysis in the Multidisciplinary Approach to the Study of Chronic Pelvic Pain (MAPP) Research Network, Neurourology and Urodynamics. (2022) 41, no. 6, 1468–1481, 10.1002/nau.24983, 35686553. 35686553 PMC11033701

[31] Place M. A. , Kelly D. , Hubbard G. , Boyd K. , Winslow F. , Leung H. , Howie C. , and Forbat L. , Prostate Cancer: Exploring the Reasons for Timing of Presentation and Diagnosis. Final Report, 2011, University of Stirling, Cancer Care Research Centre.

[32] Cuzick J. , Thorat M. A. , Andriole G. , Brawley O. W. , Brown P. H. , Culig Z. , Eeles R. A. , Ford L. G. , Hamdy F. C. , Holmberg L. , Ilic D. , Key T. J. , Vecchia C. L. , Lilja H. , Marberger M. , Meyskens F. L. , Minasian L. M. , Parker C. , Parnes H. L. , Perner S. , Rittenhouse H. , Schalken J. , Schmid H. P. , Schmitz-Dräger B. J. , Schröder F. H. , Stenzl A. , Tombal B. , Wilt T. J. , and Wolk A. , Prevention and Early Detection of Prostate Cancer, Lancet Oncology. (2014) 15, no. 11, e484–e492, 10.1016/S1470-2045(14)70211-6, 25281467. 25281467 PMC4203149

[33] Miller K. D. , Nogueira L. , Mariotto A. B. , Rowland J. H. , Yabroff K. R. , Alfano C. M. , Jemal A. , Kramer J. L. , and Siegel R. L. , Cancer Treatment and Survivorship Statistics, 2019, CA: A Cancer Journal for Clinicians. (2019) 69, no. 5, 363–385, 10.3322/caac.21565, 31184787. 31184787

[34] Chornokur G. , Dalton K. , Borysova M. E. , and Kumar N. B. , Disparities at Presentation, Diagnosis, Treatment, and Survival in African American Men, Affected by Prostate Cancer, Prostate. (2011) 71, no. 9, 985–997, 10.1002/pros.21314, 21541975. 21541975 PMC3083484

[35] Rees J. , Abrahams M. , Doble A. , Cooper A. , and Group PER , Diagnosis and Treatment of Chronic Bacterial Prostatitis and Chronic Prostatitis/Chronic Pelvic Pain Syndrome: A Consensus Guideline, BJU International. (2015) 116, no. 4, 509–525, 10.1111/bju.13101. 25711488 PMC5008168

[36] Lummus W. E. and Thompson I. , Prostatitis, Emergency Medicine Clinics of North America. (2001) 19, no. 3, 691–707, 10.1016/S0733-8627(05)70210-8. 11554282

[37] Khan F. U. , Ihsan A. U. , Khan H. U. , Jana R. , Wazir J. , Khongorzul P. , Waqar M. , and Zhou X. , Comprehensive Overview of Prostatitis, Biomedicine & Pharmacotherapy. (2017) 94, 1064–1076, 10.1016/j.biopha.2017.08.016. 28813783

[38] Murphy S. F. , Schaeffer A. J. , and Thumbikat P. , Immune Mediators of Chronic Pelvic Pain Syndrome, Nature Reviews Urology. (2014) 11, no. 5, 259–269, 10.1038/nrurol.2014.63, 24686526. 24686526 PMC4986688

[39] Oseni S. O. , Naar C. , Pavlović M. , Asghar W. , Hartmann J. X. , Fields G. B. , Esiobu N. , and Kumi-Diaka J. , The Molecular Basis and Clinical Consequences of Chronic Inflammation in Prostatic Diseases: Prostatitis, Benign Prostatic Hyperplasia, and Prostate Cancer, Cancers. (2023) 15, no. 12, 10.3390/cancers15123110, 37370720. PMC10296711 37370720

[40] Nickel J. C. , Downey J. , Hunter D. , and Clark J. , Prevalence of Prostatitis-Like Symptoms in a Population Based Study Using the National Institutes of Health Chronic Prostatitis Symptom Index, Journal of Urology. (2001) 165, no. 3, 842–845, 10.1016/S0022-5347(05)66541-X, 11176483. 11176483

[41] Repetto E. , Sosa A. , Colla R. , Revol M. , Metrebian E. , and Metrebian S. , Relationship of Prostatitis in the Appearance of Prostate Cancer and Benign Prostatic Hyperplasia, Revista Cubana de Urología. (2019) 8, no. 1, 22–33.

[42] Jiang J. , Li J. , Yunxia Z. , Zhu H. , Liu J. , and Pumill C. , The Role of Prostatitis in Prostate Cancer: Meta-Analysis, PloS One. (2013) 8, no. 12, e85179, 10.1371/journal.pone.0085179, 24391995. 24391995 PMC3877315

[43] Sfanos K. S. and De Marzo A. M. , Prostate Cancer and Inflammation: The Evidence, Histopathology. (2012) 60, no. 1, 199–215, 10.1111/j.1365-2559.2011.04033.x, 22212087. 22212087 PMC4029103

[44] Jung G. , Kim J. K. , Kim H. , Lee J. , and Hong S. K. , The Association Between Prostatitis and Risk of Prostate Cancer: A National Health Insurance Database Study, World Journal of Urology. (2022) 40, no. 11, 2781–2787, 10.1007/s00345-022-04165-2. 36201020

[45] Perletti G. , Monti E. , Magri V. , Cai T. , Cleves A. , Trinchieri A. , and Montanari E. , The Association Between Prostatitis and Prostate Cancer: Systematic Review and Meta-Analysis, Archivio Italiano di Urologia e Andrologia. (2017) 89, no. 4, 259–265, 10.4081/aiua.2017.4.259. 29473374

[46] Langston M. E. , Horn M. , Khan S. , Pakpahan R. , Doering M. , Dennis L. K. , and Sutcliffe S. , A Systematic Review and Meta-Analysis of Associations Between Clinical Prostatitis and Prostate Cancer: New Estimates Accounting for Detection Bias, Cancer Epidemiology, Biomarkers & Prevention. (2019) 28, no. 10, 1594–1603, 10.1158/1055-9965.EPI-19-0387, 31337640. PMC6774844 31337640

[47] Liu Y. , Mikrani R. , Xie D. , Wazir J. , Shrestha S. , Ullah R. , Baig M. M. F. A. , Ahmed A. , Srivastava P. K. , Thapa K. B. , and Zhou X. , Chronic Prostatitis/Chronic Pelvic Pain Syndrome And Prostate Cancer: Study of Immune Cells and Cytokines, Fundamental & Clinical Pharmacology. (2020) 34, no. 2, 160–172, 10.1111/fcp.12517, 31642541. 31642541

[48] Porcaro A. B. , Novella G. , Balzarro M. , Martignoni G. , Brunelli M. , Cacciamani G. , Cerruto M. A. , and Artibani W. , Prostate Chronic Inflammation Type IV and Prostate Cancer Risk in Patients Undergoing First Biopsy Set: Results of a Large Cohort Study, Asian Journal of Urology. (2015) 2, no. 4, 224–232, 10.1016/j.ajur.2015.08.007, 29264150. 29264150 PMC5730755

[49] Wang G. C. , Huang T. R. , Wang K. Y. , Wu Z. L. , Xie J. B. , Zhang H. L. , Yin L. , Tang W. L. , and Peng B. , Inflammation Induced by Lipopolysaccharide Advanced Androgen Receptor Expression and Epithelial-Mesenchymal Transition Progress in Prostatitis and Prostate Cancer, Translational Andrology and Urology. (2021) 10, no. 11, 4275–4287, 10.21037/tau-21-964, 34984192. 34984192 PMC8661260

[50] Devlin C. M. , Simms M. S. , and Maitland N. J. , Benign Prostatic Hyperplasia–What Do We Know?, BJU International. (2021) 127, no. 4, 389–399, 10.1111/bju.15229, 32893964. 32893964

[51] Tsunemori H. and Sugimoto M. , Effects of Inflammatory Prostatitis on the Development and Progression of Benign Prostatic Hyperplasia: A Literature Review, International Journal of Urology. (2021) 28, no. 11, 1086–1092, 10.1111/iju.14644, 34342061. 34342061

[52] Li J. , Li Y. , Cao D. , Huang Y. , Peng L. , Meng C. , and Wei Q. , The Association Between Histological Prostatitis and Benign Prostatic Hyperplasia: A Single-Center Retrospective Study, Aging Male. (2022) 25, no. 1, 95–100, 10.1080/13685538.2022.2050360, 35289705. 35289705

[53] Stewart K. L. and Lephart E. D. , Overview of BPH: Symptom Relief With Dietary Polyphenols, Vitamins and Phytochemicals by Nutraceutical Supplements With Implications to the Prostate Microbiome, International Journal of Molecular Sciences. (2023) 24, no. 6, 10.3390/ijms24065486, 36982560. PMC10058027 36982560

[54] Zhang L. , Wang Y. , Qin Z. , Gao X. , Xing Q. , Li R. , Wang W. , Song N. , and Zhang W. , Correlation Between Prostatitis, Benign Prostatic Hyperplasia and Prostate Cancer: A Systematic Review and Meta-analysis, Journal of Cancer. (2020) 11, no. 1, 177–189, 10.7150/jca.37235, 31892984. 31892984 PMC6930406

[55] Sauver J. L. , Jacobson D. J. , McGree M. E. , Girman C. J. , Lieber M. M. , and Jacobsen S. J. , Longitudinal Association Between Prostatitis and Development of Benign Prostatic Hyperplasia, Urology. (2008) 71, no. 3, 475–479, 10.1016/j.urology.2007.11.155. 18342190 PMC2597174

[56] Di Silverio F. , Gentile V. , De Matteis A. , Mariotti G. , Giuseppe V. , Luigi P. A. , and Sciarra A. , Distribution of Inflammation, Pre-Malignant Lesions, Incidental Carcinoma in Histologically Confirmed Benign Prostatic Hyperplasia: A Retrospective Analysis, European Urology. (2003) 43, no. 2, 164–175, 10.1016/S0302-2838(02)00548-1, 12565775. 12565775

[57] Wang W. , Bergh A. , and Damber J. E. , Chronic Inflammation in Benign Prostate Hyperplasia Is Associated With Focal Upregulation of Cyclooxygenase-2, Bcl-2, and Cell Proliferation in the Glandular Epithelium, Prostate. (2004) 61, no. 1, 60–72, 10.1002/pros.20061, 15287094. 15287094

[58] Shi Y. F. , Yu D. J. , Jiang C. Y. , Wang X. J. , Zhu Y. P. , Zhao R. Z. , Lv Z. , and Sun X. W. , TRAF6 Regulates Proliferation of Stromal Cells in the Transition and Peripheral Zones of Benign Prostatic Hyperplasia via Akt/mTOR Signaling, Prostate. (2018) 78, no. 3, 193–201, 10.1002/pros.23456, 29171041. 29171041

[59] Vela Navarrete R. , Garcia Cardoso J. V. , Barat A. , Manzarbeitia F. , and López F. A. , BPH and Inflammation: Pharmacological Effects of Permixon on Histological and Molecular Inflammatory Markers. Results of a Double Blind Pilot Clinical Assay, European Urology. (2003) 44, no. 5, 549–555, 10.1016/S0302-2838(03)00368-3, 14572753. 14572753

[60] Robert G. , Descazeaud A. , Nicolaïew N. , Terry S. , Sirab N. , Vacherot F. , Maillé P. , Allory Y. , and de la Taille A. , Inflammation in Benign Prostatic Hyperplasia: A 282 patients′ Immunohistochemical Analysis, Prostate. (2009) 69, no. 16, 1774–1780, 10.1002/pros.21027, 19670242. 19670242 PMC2833181

[61] Akinnuwesi B. A. , Olayanju K. A. , Aribisala B. S. , Fashoto S. G. , Mbunge E. , Okpeku M. , and Owate P. , Application of Support Vector Machine Algorithm for Early Differential Diagnosis of Prostate Cancer, Data Science and Management. (2023) 6, no. 1, 1–12, 10.1016/j.dsm.2022.10.001.

[62] Glaser A. , Shi Z. , Wei J. , Lanman N. A. , Ladson-Gary S. , Vickman R. E. , Franco O. E. , Crawford S. E. , Lilly Zheng S. , Hayward S. W. , Isaacs W. B. , Helfand B. T. , and Xu J. , Shared Inherited Genetics of Benign Prostatic Hyperplasia and Prostate Cancer, European Urology Open Science. (2022) 43, 54–61, 10.1016/j.euros.2022.07.004, 36353071. 36353071 PMC9638770

[63] Kim S. H. , Kwon W. A. , and Joung J. Y. , Impact of Benign Prostatic Hyperplasia and/or Prostatitis on the Risk of Prostate Cancer in Korean Patients, World Journal of Men′s Health. (2021) 39, no. 2, 358–365, 10.5534/wjmh.190135, 32202082. PMC7994649 32202082

[64] Ørsted D. D. , Bojesen S. E. , Nielsen S. F. , and Nordestgaard B. G. , Association of Clinical Benign Prostate Hyperplasia With Prostate Cancer Incidence and Mortality Revisited: A Nationwide Cohort Study of 3,009,258 Men, European Urology. (2011) 60, no. 4, 691–698, 10.1016/j.eururo.2011.06.016, 21705134. 21705134

[65] Hung S. C. , Lai S. W. , Tsai P. Y. , Chen P. C. , Wu H. C. , Lin W. H. , and Sung F. C. , Synergistic Interaction of Benign Prostatic Hyperplasia and Prostatitis on Prostate Cancer Risk, British Journal of Cancer. (2013) 108, no. 9, 1778–1783, 10.1038/bjc.2013.184, 23612451. 23612451 PMC3658521

[66] Miah S. and Catto J. , BPH and Prostate Cancer Risk, Indian Journal of Urology. (2014) 30, no. 2, 214–218, 10.4103/0970-1591.126909, 24744523. 24744523 PMC3989826

[67] Schenk J. M. , Kristal A. R. , Arnold K. B. , Tangen C. M. , Neuhouser M. L. , Lin D. W. , White E. , and Thompson I. M. , Association of Symptomatic Benign Prostatic Hyperplasia and Prostate Cancer: Results From the Prostate Cancer Prevention Trial, American Journal of Epidemiology. (2011) 173, no. 12, 1419–1428, 10.1093/aje/kwq493, 21540324. 21540324 PMC3276227

[68] Chokkalingam A. P. , Nyrén O. , Johansson J. E. , Gridley G. , McLaughlin J. K. , Adami H. O. , and Hsing A. W. , Prostate Carcinoma Risk Subsequent to Diagnosis of Benign Prostatic Hyperplasia, Cancer: Interdisciplinary International Journal of the American Cancer Society. (2003) 98, no. 8, 1727–1734, 10.1002/cncr.11710. 14534890

[69] He J. , Han Z. , Luo W. , Shen J. , Xie F. , Liao L. , Zou G. , Luo X. , Guo Z. , Li Y. , Li J. , and Chen H. , Serum Organic Acid Metabolites Can Be Used as Potential Biomarkers to Identify Prostatitis, Benign Prostatic Hyperplasia, and Prostate Cancer, Frontiers in Immunology. (2023) 13, 998447, 10.3389/fimmu.2022.998447, 36685547. 36685547 PMC9846500

[70] Mancuso G. , Midiri A. , Gerace E. , Marra M. , Zummo S. , and Biondo C. , Urinary Tract Infections: The Current Scenario and Future Prospects, Pathogens. (2023) 12, no. 4, 10.3390/pathogens12040623, 37111509. PMC10145414 37111509

[71] Rahimzadeh M. , Shahbazi S. , Sabzi S. , Habibi M. , and Asadi Karam M. R. , Antibiotic Resistance and Genetic Diversity AmongPseudomonas AeruginosaIsolated From Urinary Tract Infections in Iran, Future Microbiology. (2023) 18, no. 16, 1171–1183, 10.2217/fmb-2023-0118. 37882782

[72] Pothoven R. , Management of Urinary Tract Infections in the Era of Antimicrobial Resistance, Drug Target Insights. (2023) 17, 126–137, 10.33393/dti.2023.2660, 38124759. 38124759 PMC10731245

[73] Whelan S. , Lucey B. , and Finn K. , Uropathogenic Escherichia coli (UPEC)-Associated Urinary Tract Infections: The Molecular Basis for Challenges to Effective Treatment, Microorganisms. (2023) 11, no. 9, 10.3390/microorganisms11092169, 37764013. PMC10537683 37764013

[74] Lee T. , Pang S. , Abraham S. , and Coombs G. W. , Antimicrobial-Resistant CC17 Enterococcus faecium: The Past, the Present and the Future, Journal of Global Antimicrobial Resistance. (2019) 16, 36–47, 10.1016/j.jgar.2018.08.016, 30149193. 30149193

[75] Sabzi S. , Mashhadi R. , and Pourmand M. R. , Fibrinogen and Mucin Binding Activity of EF0737, a Novel Protein of Enterococcus faecalis, Iranian Journal of Microbiology. (2017) 9, no. 6, 324–330, 29487730. 29487730 PMC5825932

[76] Shivaee A. and Mirshekar M. , Association Between ESBLs Genes and Quinolone Resistance in Uropathogenic Escherichia coli Isolated From Patients With Urinary Tract Infection, Infection Epidemiology and Microbiology. (2019) 5, no. 1, 15–23.

[77] Storme O. , Tirán Saucedo J. , Garcia-Mora A. , Dehesa-Dávila M. , and Naber K. G. , Risk Factors and Predisposing Conditions for Urinary Tract Infection, Therapeutic Advances in Urology. (2019) 11, 1756287218814382, 10.1177/1756287218814382, 31105772. 31105772 PMC6502981

[78] Boehm K. , Valdivieso R. , Meskawi M. , Larcher A. , Schiffmann J. , Sun M. , Graefen M. , Saad F. , Parent M. É. , and Karakiewicz P. I. , Prostatitis, Other Genitourinary Infections and Prostate Cancer: Results From a Population-Based Case–Control Study, World Journal of Urology. (2016) 34, no. 3, 425–430, 10.1007/s00345-015-1625-1, 26108732. 26108732

[79] Lipsky B. A. , Byren I. , and Hoey C. T. , Treatment of Bacterial Prostatitis, Clinical Infectious Diseases. (2010) 50, no. 12, 1641–1652, 10.1086/652861. 20459324

[80] Naber K. , Roscher K. , Botto H. , and Schaefer V. , Oral Levofloxacin 500mg Once Daily in the Treatment of Chronic Bacterial Prostatitis, International Journal of Antimicrobial Agents. (2008) 32, no. 2, 145–153, 10.1016/j.ijantimicag.2008.03.014. 18571904

[81] Landau A. and Welliver C. , Analyzing and Characterizing Why Men Seek Care for Lower Urinary Tract Symptoms, Current Urology Reports. (2020) 21, no. 12, 1–5, 10.1007/s11934-020-01006-w. 33128142

[82] Irwin D. E. , Milsom I. , Hunskaar S. , Reilly K. , Kopp Z. , Herschorn S. , Coyne K. , Kelleher C. , Hampel C. , Artibani W. , and Abrams P. , Population-Based Survey of Urinary Incontinence, Overactive Bladder, and Other Lower Urinary Tract Symptoms in Five Countries: Results of the EPIC Study, European Urology. (2006) 50, no. 6, 1306–1315, 10.1016/j.eururo.2006.09.019, 17049716. 17049716

[83] Magistro G. , Keller P. , Westhofen T. , Schott M. , Tamalunas A. , Weinhold P. , and Stief C. G. , The Significance of a High Preoperative PSA Level for the Detection of Incidental Prostate Cancer in LUTS Patients With Large Prostates, World Journal of Urology. (2021) 39, no. 5, 1481–1487, 10.1007/s00345-020-03321-w, 32588205. 32588205

[84] Zhang T. , Wu H. , Liu S. , He W. , and Ding K. , Clinical Evaluation of Tamsulosin in the Relief of Lower Urinary Tract Symptoms in Advanced Prostate Cancer Patients, International Urology and Nephrology. (2017) 49, no. 7, 1111–1117, 10.1007/s11255-017-1591-1, 28409402. 28409402

[85] Wu M. , Pan C. , He Y. , and Yang B. , A Novel Nomogram for Identifying the Patients at Risk for Rapid Progression of Advanced Hormone-Sensitive Prostate Cancer, Cancer Management and Research. (2023) 15, 1015–1024, 10.2147/CMAR.S425181, 37746314. 37746314 PMC10516215

[86] Koo M. M. , Swann R. , McPhail S. , Abel G. A. , Elliss-Brookes L. , Rubin G. P. , and Lyratzopoulos G. , Presenting Symptoms of Cancer and Stage at Diagnosis: Evidence From a Cross-Sectional, Population-Based Study, Lancet Oncology. (2020) 21, no. 1, 73–79, 10.1016/S1470-2045(19)30595-9, 31704137. 31704137 PMC6941215

[87] Rains A. and Ritchie H. D. , Bailey & Love’s Short Textbook of Surgery, 1984, HK Lewis.

[88] Frånlund M. , Carlsson S. , Stranne J. , Aus G. , and Hugosson J. , The Absence of Voiding Symptoms in Men With a Prostate-Specific Antigen (PSA) Concentration of≥ 3.0 ng/mL Is an Independent Risk Factor for Prostate Cancer: Results From the Gothenburg Randomized Screening Trial, BJU International. (2012) 110, no. 5, 638–643, 10.1111/j.1464-410X.2012.10962.x, 22540895. 22540895 PMC5629001

[89] Al-Zubaidi M. , Hawks C. , Fernando S. , Moe A. , Swarbrick N. , McCombie S. P. , Brown M. , and Hayne D. , Relationship Between Lower Urinary Tract Symptoms (LUTS) and Prostate Cancer: A Persistent Myth, Urology. (2025) 18, no. 2, 129–136, 10.1177/20514158231170420.

[90] Chandra Engel J. , Palsdottir T. , Aly M. , Egevad L. , Grönberg H. , Eklund M. , and Nordström T. , Lower Urinary Tract Symptoms (LUTS) Are Not Associated With an Increased Risk of Prostate Cancer in Men 50–69 Years With PSA≥ 3 ng/ml, Scandinavian Journal of Urology. (2020) 54, no. 1, 1–6, 10.1080/21681805.2019.1703806. 31876229

[91] Ward J. and Girgis S. , GPs′ Estimates of Men′s Risk of Prostate Cancer and Screening Expectations, Australian and New Zealand Journal of Public Health. (1999) 23, no. 2, 219–220, 10.1111/j.1467-842X.1999.tb01243.x, 10330746. 10330746

[92] Russell B. , Garmo H. , Beckmann K. , Stattin P. , Adolfsson J. , and Van Hemelrijck M. , A Case-Control Study of Lower Urinary-Tract Infections, Associated Antibiotics and the Risk of Developing Prostate Cancer Using PCBaSe 3.0, PloS One. (2018) 13, no. 4, e0195690, 10.1371/journal.pone.0195690, 29649268. 29649268 PMC5896993

[93] Huang C.-H. , Chou Y.-H. , Yeh H.-W. , Huang J.-Y. , Yang S.-F. , and Yeh C.-B. , Risk of Cancer After Lower Urinary Tract Infection: A Population-Based Cohort Study, International Journal of Environmental Research and Public Health. (2019) 16, no. 3, 10.3390/ijerph16030390, 30704106. PMC6388119 30704106

[94] Tolani M. A. , Suleiman A. , Awaisu M. , Abdulaziz M. M. , Lawal A. T. , and Bello A. , Acute Urinary Tract Infection in Patients With Underlying Benign Prostatic Hyperplasia and Prostate Cancer, Pan African Medical Journal. (2020) 36, no. 1, 10.11604/pamj.2020.36.169.21038, 32952813. PMC7467887 32952813

[95] Yin S. , Xu D. , Zhang M. , Zhang P. , Guan Y. , Kzhyshkowska J. , and Liang C. , Urine Flora Imbalance and New Biomarkers in Prostate Cancer and Benign Prostatic Hyperplasia, Archives of Medical Science. (2021) 4, 1–11, 10.5114/aoms/135380.

[96] Pan S.-Y. , Chen W.-C. , Huang C.-P. , Hsu C. Y. , and Chang Y.-H. , The Association of Prostate Cancer and Urinary Tract Infections: A New Perspective of Prostate Cancer Pathogenesis, Medicina. (2023) 59, no. 3, 10.3390/medicina59030483, 36984484. PMC10056160 36984484

[97] Hyun J. , Ha M. S. , Oh S. Y. , Tae J. H. , Chi B. H. , Chang I. H. , Kim T. H. , Myung S. C. , Nguyen T. T. , Kim J. H. , Kim J. W. , Lee Y. S. , Lee J. , and Choi S. Y. , Urinary Tract Infection After Radiation Therapy or Radical Prostatectomy on the Prognosis of Patients With Prostate Cancer: A Population-Based Study, BMC Cancer. (2023) 23, no. 1, 10.1186/s12885-023-10869-4, 37138203. PMC10157974 37138203

[98] Gonçalves M. F. , Pina-Vaz T. , Fernandes Â. R. , Miranda I. M. , Silva C. M. , Rodrigues A. G. , and Lisboa C. , Microbiota of Urine, Glans and Prostate Biopsies in Patients With Prostate Cancer Reveals a Dysbiosis in the Genitourinary System, Cancers. (2023) 15, no. 5, 10.3390/cancers15051423, 36900215. PMC10000660 36900215

[99] Örtegren J. , Kohestani K. , Elvstam O. , Janson H. , Åberg D. , Kjölhede H. , Kahlmeter G. , and Bratt O. , Risk Factors for Infection After Transrectal Prostate Biopsy: A Population-Based Register Study, European Urology Open Science. (2024) 67, 1–6, 10.1016/j.euros.2024.06.015, 39104794. 39104794 PMC11298891

[100] Al-Karawi A. S. , Abid F. M. , Mustafa A. , and Abdulla M. , Revealing the Urinary Microbiota in Prostate Cancer: A Comprehensive Review Unveiling Insights Into Pathogenesis and Clinical Application, Al-Salam Journal for Medical Science. (2023) 3, no. 1, 45–54, 10.55145/ajbms.2024.03.01.008.

[101] Randazzo G. , Bovolenta E. , Ceccato T. , Reitano G. , Betto G. , Novara G. , Iafrate M. , Morlacco A. , Dal Moro F. , and Zattoni F. , Urinary Microbiome and Urological Cancers: A Mini Review, Frontiers in Urology. (2024) 4, 1367720, 10.3389/fruro.2024.1367720, 40777102. 40777102 PMC12327262

[102] Ruplin A. , Segal E. , and McFarlane T. , Review of Drug-Drug Interactions in Patients With Prostate Cancer, Journal of Oncology Pharmacy Practice. (2024) 30, no. 6, 1057–1072, 10.1177/10781552241238198, 38720547. 38720547 PMC11476483

[103] Elkrief A. , Derosa L. , Kroemer G. , Zitvogel L. , and Routy B. , The Negative Impact of Antibiotics on Outcomes in Cancer Patients Treated With Immunotherapy: A New Independent Prognostic Factor?, Annals of Oncology. (2019) 30, no. 10, 1572–1579, 10.1093/annonc/mdz206, 31268133. 31268133

[104] Derosa L. , Hellmann M. D. , Spaziano M. , Halpenny D. , Fidelle M. , Rizvi H. , Long N. , Plodkowski A. J. , Arbour K. C. , Chaft J. E. , Rouche J. A. , Zitvogel L. , Zalcman G. , Albiges L. , Escudier B. , and Routy B. , Negative Association of Antibiotics on Clinical Activity of Immune Checkpoint Inhibitors in Patients With Advanced Renal Cell and Non-Small-Cell Lung Cancer, Annals of Oncology. (2018) 29, no. 6, 1437–1444, 10.1093/annonc/mdy103, 29617710. 29617710 PMC6354674

[105] Tien N. , Lin T.-H. , Hung Z.-C. , Lin H.-S. , Wang I.-K. , Chen H.-C. , and Chang C. T. , Diagnosis of Bacterial Pathogens in the Urine of Urinary-Tract-Infection Patients Using Surface-Enhanced Raman Spectroscopy, Molecules. (2018) 23, no. 12, 10.3390/molecules23123374, 30572659. PMC6321215 30572659

[106] Nassaji M. , Ghorbani R. , and Afshar R. K. , The Effect of Statins Use on the Risk and Outcome of Acute Bacterial Infections in Adult Patients, Journal of Clinical and Diagnostic Research. (2015) 9, no. 11, 10.7860/JCDR/2015/14538.6773. PMC4668448 26676277

[107] Fitzpatrick J. M. , Desgrandchamps F. , Adjali K. , Guerra L. G. , Hong S. J. , Khalid S. E. , Ratana-Olarn K. , and for the Reten-World Study Group , Management of Acute Urinary Retention: A Worldwide Survey of 6074 Men With Benign Prostatic Hyperplasia, BJU International. (2012) 109, no. 1, 88–95, 10.1111/j.1464-410X.2011.10430.x, 22117624. 22117624 PMC3272343

[108] Antunes A. A. , Barbosa J. A. B. , Reis S. T. , Guariero M. S. , Fukushima J. T. , Dall′Oglio M. F. , Freire G. D. , Lucon A. M. , Leite K. R. , and Srougi M. , Prostate Biopsy in Patients With Long-Term Use of Indwelling Bladder Catheter: What Is the Rationale?, Urologic Oncology: Seminars and Original Investigations. (2012) 30, no. 5, 583–586, 10.1016/j.urolonc.2010.06.012. 20933446

[109] Kravchick S. , Bunkin I. , Peled R. , Yulish E. , Ben-Dor D. , Kravchenko Y. , and Cytron S. , Patients With Elevated Serum PSA and Indwelling Catheter After Acute Urinary Retention: Prospective Study of 63 Patients With 7-Year Follow-Up, Journal of Endourology. (2007) 21, no. 10, 1203–1206, 10.1089/end.2007.9907, 17949326. 17949326

[110] Izadpanahi M. H. , Salimi H. , Javid A. , and Eslami S. , The Effect of Urethral Catheterization on the Level of Prostate-Specific Antigen, Journal of Research in Medical Sciences: The Official Journal of Isfahan University of Medical Sciences. (2017) 22, no. 1, 10.4103/1735-1995.202145. PMC5393098 28465697

[111] Stephan C. , Ralla B. , and Jung K. , Prostate-Specific Antigen and Other Serum and Urine Markers in Prostate Cancer, Biochimica et Biophysica Acta (BBA)-Reviews on Cancer. (2014) 1846, no. 1, 99–112, 10.1016/j.bbcan.2014.04.001. 24727384

[112] Batislam E. , Arik A. I. , Karakoc A. , Uygur M. C. , Germiyanoğlu R. C. , and Erol D. , Effect of Transurethral Indwelling Catheter on Serum Prostate-Specific Antigen Level in Benign Prostatic Hyperplasia, Urology. (1997) 49, no. 1, 50–54, 10.1016/S0090-4295(96)00386-X, 9000185. 9000185

[113] Bushman W. , Etiology, Epidemiology, and Natural History, Urologic Clinics. (2009) 36, no. 4, 403–415, 10.1016/j.ucl.2009.07.003. 19942041

[114] Iqbal M. , Khurshid M. A. , Hassan S. , Naeem M. A. , Niaz S. , and Siddiqui A. A. , Effect of Different Manipulations on Serum PSA in Patients With Benign Prostatic Hyperplasia, Professional Medical Journal. (2021) 28, no. 6, 866–871, 10.29309/TPMJ/2021.28.06.5990.

[115] Matzkin H. , Laufer M. , Chen J. , Hareuveni M. , and Braf Z. , Effect of Elective Prolonged Urethral Catheterization on Serum Prostate-Specific Antigen Concentration, Urology. (1996) 48, no. 1, 63–66, 10.1016/S0090-4295(96)00087-8, 8693653. 8693653

[116] Aslan Y. , Tuncel A. , Tekdoğan Ü. , Oezergin O. , and Atan A. , Is Serum Prostate Specific Antigen Level Affected by In-and-Out Urethral Catheterization?, Turkish Journal of Medical Sciences. (2007) 37, no. 4, 195–198.

[117] Hacıislamoğlu A. , Evren İ. , Yavuzsan A. H. , Yiğitbaşı İ. , Ekşi M. , Şeker K. G. , and Tuğcu V. , Role of Prostate Specific Antijen in Prostatic Adenocarcinoma Detection in Patients With Benign Prostate Hyperplasia and Were Placed Urethral Catheter After Acute Urinary Retention, Yeni Üroloji Dergisi. (2020) 15, no. 3, 158–163.

[118] Faris G. , Levi S. , Teib H. , Friedman M. , Shotland Y. , Zivan I. , and Stein A. , IsPSAReliable in Patients With Indwelling Urethral Catheter?, UroOncology. (2003) 2, no. 3, 117–120, 10.1080/1561095021000018687.

[119] Bharti S. V. , Correlation Between Serum Prostatic Specific Antigen and Prostatic Volume in Benign Prostatic Hyperplasia, Journal of Nepalgunj Medical College. (2017) 15, no. 1, 9–15.

[120] Yebes A. , Toribio-Vazquez C. , Martinez-Perez S. , Quesada-Olarte J. , Rodriguez-Serrano A. , Álvarez-Maestro M. , and Martinez-Piñeiro L. , Prostatitis: A Review, Current Urology Reports. (2023) 24, no. 5, 241–251, 10.1007/s11934-023-01150-z. 36881349

[121] Platz E. A. , Kulac I. , Barber J. R. , Drake C. G. , Joshu C. E. , Nelson W. G. , Lucia M. S. , Klein E. A. , Lippman S. M. , Parnes H. L. , Thompson I. M. , Goodman P. J. , Tangen C. M. , and de Marzo A. M. , A Prospective Study of Chronic Inflammation in Benign Prostate Tissue and Risk of Prostate Cancer: Linked PCPT and SELECT Cohorts, Cancer Epidemiology, Biomarkers & Prevention. (2017) 26, no. 10, 1549–1557, 10.1158/1055-9965.EPI-17-0503, 28754796. PMC5626618 28754796

[122] Zhang Y. , Zhou C. K. , Rencsok E. M. , Fall K. , Lotan T. L. , Loda M. , Giunchi F. , Platz E. A. , de Marzo A. M. , Mucci L. A. , Fiorentino M. , and Ebot E. M. , A prospective Study of Intraprostatic Inflammation, Focal Atrophy, and Progression to Lethal Prostate Cancer, Cancer Epidemiology, Biomarkers & Prevention. (2019) 28, no. 12, 2047–2054, 10.1158/1055-9965.EPI-19-0713, 31533941. PMC6941930 31533941

[123] Cavarretta I. , Ferrarese R. , Cazzaniga W. , Saita D. , Lucianò R. , Ceresola E. R. , Locatelli I. , Visconti L. , Lavorgna G. , Briganti A. , Nebuloni M. , Doglioni C. , Clementi M. , Montorsi F. , Canducci F. , and Salonia A. , The Microbiome of the Prostate Tumor Microenvironment, European Urology. (2017) 72, no. 4, 625–631, 10.1016/j.eururo.2017.03.029, 28434677. 28434677

[124] Shinohara D. B. , Vaghasia A. M. , Yu S. H. , Mak T. N. , Brüggemann H. , Nelson W. G. , de Marzo A. M. , Yegnasubramanian S. , and Sfanos K. S. , A Mouse Model of Chronic Prostatic Inflammation Using a Human Prostate Cancer-Derived Isolate of Propionibacterium acnes, Prostate. (2013) 73, no. 9, 1007–1015, 10.1002/pros.22648, 23389852. 23389852 PMC3991131

[125] Fehri L. F. , Mak T. N. , Laube B. , Brinkmann V. , Ogilvie L. A. , Mollenkopf H. , Lein M. , Schmidt T. , Meyer T. F. , and Brüggemann H. , Prevalence of Propionibacterium Acnes in Diseased Prostates and Its Inflammatory and Transforming Activity on Prostate Epithelial Cells, International Journal of Medical Microbiology. (2011) 301, no. 1, 69–78, 10.1016/j.ijmm.2010.08.014, 20943438. 20943438

[126] Shannon B. A. , Garrett K. L. , and Cohen R. J. , Links Between *Propionibacterium acnes* and Prostate Cancer, Future Oncology. (2006) 2, no. 2, 225–232, 10.2217/14796694.2.2.225, 16563091. 16563091

[127] Sutcliffe S. , Giovannucci E. , Isaacs W. B. , Willett W. C. , and Platz E. A. , Acne and Risk of Prostate Cancer, International Journal of Cancer. (2007) 121, no. 12, 2688–2692, 10.1002/ijc.23032, 17724724. 17724724 PMC3076591

[128] Hernández-Pérez J. G. , López D. S. , Rodríguez-Valentín R. , Vázquez-Salas R. A. , Sierra-Santoyo A. , and Torres-Sánchez L. , Late Puberty Onset and Lack of Acne During Adolescence Reduce High-Grade Prostate Cancer at Adulthood, Prostate. (2023) 83, no. 14, 1342–1350, 10.1002/pros.24596, 37415324. 37415324

[129] Galobardes B. , Davey Smith G. , Jeffreys M. , Kinra S. , and McCarron P. , Acne in Adolescence and Cause-Specific Mortality: Lower Coronary Heart Disease but Higher Prostate Cancer Mortality: The Glasgow Alumni Cohort Study, American Journal of Epidemiology. (2005) 161, no. 12, 1094–1101, 10.1093/aje/kwi147, 15937017. 15937017

[130] Ugge H. , Udumyan R. , Carlsson J. , Andrén O. , Montgomery S. , Davidsson S. , and Fall K. , Acne in Late Adolescence and Risk of Prostate Cancer, International Journal of Cancer. (2018) 142, no. 8, 1580–1585, 10.1002/ijc.31192, 29205339. 29205339 PMC5838533

[131] McLaughlin J. , Watterson S. , Layton A. M. , Bjourson A. J. , Barnard E. , and McDowell A. , Propionibacterium acnes and Acne Vulgaris: New Insights From the Integration of Population Genetic, Multi-Omic, Biochemical and Host-Microbe Studies, Microorganisms. (2019) 7, no. 5, 10.3390/microorganisms7050128, 31086023. PMC6560440 31086023

[132] Davidsson S. , Mölling P. , Rider J. R. , Unemo M. , Karlsson M. G. , Carlsson J. , Andersson S. O. , Elgh F. , Söderquist B. , and Andrén O. , Frequency and Typing of *Propionibacterium acnes* in Prostate Tissue Obtained From Men With and Without Prostate Cancer, Infectious Agents and Cancer. (2016) 11, no. 1, 10.1186/s13027-016-0074-9, 27284286. PMC4899914 27284286

[133] Feng Y. , Jaratlerdsiri W. , Patrick S. M. , Lyons R. J. , Haynes A. M. , Collins C. C. , Stricker P. D. , Bornman M. S. R. , and Hayes V. M. , Metagenomic Analysis Reveals a Rich Bacterial Content in High-Risk Prostate Tumors From African Men, Prostate. (2019) 79, no. 15, 1731–1738, 10.1002/pros.23897, 31454437. 31454437 PMC6790596

[134] Banerjee S. , Alwine J. C. , Wei Z. , Tian T. , Shih N. , Sperling C. , Guzzo T. , Feldman M. D. , and Robertson E. S. , Microbiome Signatures in Prostate Cancer, Carcinogenesis. (2019) 40, no. 6, 749–764, 10.1093/carcin/bgz008. 30794288

[135] Sarkar P. , Malik S. , Banerjee A. , Datta C. , Pal D. K. , Ghosh A. , and Saha A. , Differential Microbial Signature Associated With Benign Prostatic Hyperplasia and Prostate Cancer, Frontiers in Cellular and Infection Microbiology. (2022) 12, 894777, 10.3389/fcimb.2022.894777, 35865814. 35865814 PMC9294280

[136] Salachan P. V. , Rasmussen M. , Fredsøe J. , Ulhøi B. , Borre M. , and Sørensen K. D. , Microbiota of the Prostate Tumor Environment Investigated by Whole-Transcriptome Profiling, Genome Medicine. (2022) 14, no. 1, 10.1186/s13073-022-01011-3, 35078527. PMC8787950 35078527

[137] Ma J. , Gnanasekar A. , Lee A. , Li W. T. , Haas M. , Wang-Rodriguez J. , Chang E. Y. , Rajasekaran M. , and Ongkeko W. M. , Influence of Intratumor Microbiome on Clinical Outcome and Immune Processes in Prostate Cancer, Cancers. (2020) 12, no. 9, 10.3390/cancers12092524, 32899474. PMC7564876 32899474

[138] Caini S. , Gandini S. , Dudas M. , Bremer V. , Severi E. , and Gherasim A. , Sexually Transmitted Infections and Prostate Cancer Risk: A Systematic Review and Meta-Analysis, Cancer Epidemiology. (2014) 38, no. 4, 329–338, 10.1016/j.canep.2014.06.002, 24986642. 24986642

[139] Taylor M. L. , Mainous A. , and Wells B. J. , Prostate Cancer and Sexually Transmitted Diseases: A Meta-Analysis, Family Medicine-Kansas City. (2005) 37, no. 7. 15988645

[140] Lawson J. S. and Glenn W. K. , Multiple Pathogens and Prostate Cancer, Infectious Agents and Cancer. (2022) 17, no. 1. 10.1186/s13027-022-00427-1 PMC9150368 35637508

[141] Ravich A. and Ravich R. , Prophylaxis of Cancer of the Prostate, Penis, and Cervix by Circumcision, New York State Journal of Medicine. (1951) 51, no. 12, 1519–1520, 14853120. 14853120

[142] Garbas K. , Zapała P. , Zapała Ł. , and Radziszewski P. , The Role of Microbial Factors in Prostate Cancer Development—An Up-to-Date Review, Journal of Clinical Medicine. (2021) 10, no. 20, 10.3390/jcm10204772, 34682893. PMC8538262 34682893

[143] Lawson J. S. and Glenn W. K. , Evidence for a Causal Role by Human Papillomaviruses in Prostate Cancer – A Systematic Review, Infectious Agents and Cancer. (2020) 15, no. 1, 10.1186/s13027-020-00305-8, 32684946. PMC7359253 32684946

[144] Glenn W. K. , Ngan C. C. , Amos T. G. , Edwards R. J. , Swift J. , Lutze-Mann L. , Shang F. , Whitaker N. J. , and Lawson J. S. , High Risk Human Papilloma Viruses (HPVs) Are Present in Benign Prostate Tissues Before Development of HPV Associated Prostate Cancer, Infectious Agents and Cancer. (2017) 12, no. 1, 10.1186/s13027-017-0157-2, 28811834. PMC5553674 28811834

[145] Schütze D. M. , Snijders P. J. , Bosch L. , Kramer D. , Meijer C. J. , and Steenbergen R. D. , Differential *In Vitro* Immortalization Capacity of Eleven (Probable) [Corrected] High-Risk Human Papillomavirus Types, Journal of Virology. (2014) 88, no. 3, 1714–1724, 10.1128/JVI.02859-13, 24257607. 24257607 PMC3911618

[146] Patra S. , Shand H. , Ghosal S. , and Ghorai S. , HPV and Male Cancer: Pathogenesis, Prevention and Impact, Journal of the Oman Medical Association. (2025) 2, no. 1, 10.3390/joma2010004.

[147] Fong Amaris W. M. , de Assumpção P. P. , Valadares L. J. , and Moreira F. C. , Microbiota Changes: The Unseen Players in Cervical Cancer Progression, Frontiers in Microbiology. (2024) 15, 1352778, 10.3389/fmicb.2024.1352778, 38389527. 38389527 PMC10881787

[148] Kim S. J. , Park M. , Choi A. , and Yoo S. , Microbiome and Prostate Cancer: Emerging Diagnostic and Therapeutic Opportunities, Pharmaceuticals. (2024) 17, no. 1, 10.3390/ph17010112, 38256945. PMC10819128 38256945

[149] Unemo M. , Seifert H. S. , Hook E. W. , Hawkes S. , Ndowa F. , and Dillon J. A. R. , Gonorrhoea, Nature Reviews Disease Primers. (2019) 5, no. 1, 10.1038/s41572-019-0128-6. 31754194

[150] Belcher T. , Rollier C. S. , Dold C. , Ross J. D. C. , and MacLennan C. A. , Immune Responses to *Neisseria gonorrhoeae* and Implications for Vaccine Development, Frontiers in Immunology. (2023) 14, 1248613, 10.3389/fimmu.2023.1248613, 37662926. 37662926 PMC10470030

[151] Lian W. Q. , Luo F. , Song X. L. , Lu Y. J. , and Zhao S. C. , Gonorrhea and Prostate Cancer Incidence: An Updated Meta-Analysis of 21 Epidemiologic Studies, Medical Science Monitor: International Medical Journal of Experimental and Clinical Research. (2015) 21, 1902–1910, 10.12659/MSM.893579, 26126881. 26126881 PMC4502545

[152] Johnston C. and Corey L. , Current Concepts for Genital Herpes Simplex Virus Infection: Diagnostics and Pathogenesis of Genital Tract Shedding, Clinical Microbiology Reviews. (2016) 29, no. 1, 149–161, 10.1128/CMR.00043-15, 26561565. 26561565 PMC4771215

[153] Tronstein E. , Johnston C. , Huang M. L. , Selke S. , Magaret A. , Warren T. , Corey L. , and Wald A. , Genital Shedding of Herpes Simplex Virus Among Symptomatic and Asymptomatic Persons With HSV-2 Infection, JAMA. (2011) 305, no. 14, 1441–1449, 10.1001/jama.2011.420, 21486977. 21486977 PMC3144252

[154] Parra-Sánchez M. , Genital Ulcers Caused by Herpes Simplex Virus, Enfermedades Infecciosas y Microbiologia Clinica. (2019) 37, no. 4, 260–264, 10.1016/j.eimc.2018.10.020, 30580877. 30580877

[155] Huang W.-Y. , Hayes R. , Pfeiffer R. , Viscidi R. P. , Lee F. K. , Wang Y. F. , Reding D. , Whitby D. , Papp J. R. , and Rabkin C. S. , Sexually Transmissible Infections and Prostate Cancer Risk, Cancer Epidemiology Biomarkers & Prevention. (2008) 17, no. 9, 2374–2381, 10.1158/1055-9965.EPI-08-0173, 18768506. PMC2559953 18768506

[156] Dennis L. K. , Coughlin J. A. , McKinnon B. C. , Wells T. S. , Gaydos C. A. , Hamsikova E. , and Gray G. C. , Sexually Transmitted Infections and Prostate Cancer Among Men in the U.S. Military, Cancer Epidemiology, Biomarkers & Prevention. (2009) 18, no. 10, 2665–2671, 10.1158/1055-9965.EPI-08-1167, 19755645. 19755645

[157] Bergh J. , Marklund I. , Gustavsson C. , Wiklund F. , Grönberg H. , Allard A. , Alexeyev O. , and Elgh F. , No Link Between Viral Findings in the Prostate and Subsequent Cancer Development, British Journal of Cancer. (2007) 96, no. 1, 137–139, 10.1038/sj.bjc.6603480, 17117176. 17117176 PMC2360195

[158] Sfanos K. S. , Isaacs W. B. , and De Marzo A. M. , Infections and Inflammation in Prostate Cancer, American Journal of Clinical and Experimental Urology. (2013) 1, no. 1, 3–11, 25110720. 25110720 PMC4219279

[159] Tantengco O. A. G. , Aquino I. M. C. , de Castro S. M. , Rojo R. D. , and Abad C. L. R. , Association of Mycoplasma With Prostate Cancer: A Systematic Review and Meta-Analysis, Cancer Epidemiology. (2021) 75, 102021, 10.1016/j.canep.2021.102021, 34517226. 34517226

[160] Hrbacek J. , Urban M. , Hamsikova E. , Tachezy R. , Eis V. , Brabec M. , and Heracek J. , Serum Antibodies Against Genitourinary Infectious Agents in Prostate Cancer and Benign Prostate Hyperplasia Patients: A Case-Control Study, BMC Cancer. (2011) 11, no. 1, 10.1186/1471-2407-11-53. PMC3039631 21291519

[161] Lumme S. , Tenkanen L. , Langseth H. , Gislefoss R. , Hakama M. , Stattin P. , Hallmans G. , Adlercreutz H. , Saikku P. , Stenman U. H. , Tuohimaa P. , Luostarinen T. , and Dillner J. , Longitudinal Biobanks-Based Study on the Joint Effects of Infections, Nutrition and Hormones on Risk of Prostate Cancer, Acta Oncologica. (2016) 55, no. 7, 839–845, 10.3109/0284186X.2016.1139178, 26878091. 26878091

[162] Barykova Y. A. , Logunov D. Y. , Shmarov M. M. , Vinarov A. Z. , Fiev D. N. , Vinarova N. A. , Rakovskaya I. V. , Baker P. S. , Shyshynova I. , Stephenson A. J. , Klein E. A. , Naroditsky B. S. , Gintsburg A. L. , and Gudkov A. V. , Association of Mycoplasma hominis Infection With Prostate Cancer, Oncotarget. (2011) 2, no. 4, 289–297, 10.18632/oncotarget.256, 21471611. 21471611 PMC3248169

[163] Saadat S. , Karami P. , Jafari M. , Kholoujini M. , Rikhtegaran Tehrani Z. , Mohammadi Y. , and Alikhani M. Y. , The Silent Presence of *Mycoplasma hominis* in Patients With Prostate Cancer, Pathogens and Disease. (2020) 78, no. 7, ftaa037, 10.1093/femspd/ftaa037. 32940669

[164] Erturhan S. M. , Bayrak O. , Pehlivan S. , Ozgul H. , Seckiner I. , Sever T. , and Karakök M. , Can Mycoplasma Contribute to Formation of Prostate Cancer?, International Urology and Nephrology. (2013) 45, no. 1, 33–38, 10.1007/s11255-012-0299-5, 23001641. 23001641

[165] Edwards T. , Burke P. , Smalley H. , and Hobbs G. , *Trichomonas vaginalis*: Clinical Relevance, Pathogenicity and Diagnosis, Critical Reviews in Microbiology. (2016) 42, no. 3, 406–417, 10.3109/1040841X.2014.958050, 25383648. 25383648

[166] Yadav S. , Yadav A. , and Mishra R. K. , Khare P. and Jain A. , Chapter 7 - Trichomonas vaginalis Infection and Male Reproductive Health, Trichomonas Vaginalis, 2025, Academic Press, 111–129, 10.1016/B978-0-443-22204-7.00007-1.

[167] Saleh N. E. , Alhusseiny S. M. , El-Zayady W. M. , Aboelnaga E. M. , El-Beshbishi W. N. , Saleh Y. M. , Abou-ElWafa H. S. , and El-Beshbishi S. N. , *Trichomonas vaginalis* Serostatus and Prostate Cancer Risk in Egypt: A Case-Control Study, Parasitology Research. (2021) 120, no. 4, 1379–1388, 10.1007/s00436-020-06942-7, 33159459. 33159459

[168] Kim J. H. , Moon H. S. , Kim K. S. , Hwang H. S. , Ryu J. S. , and Park S. Y. , Comparison of Seropositivity to Trichomonas vaginalis Between Men With Prostatic Tumor and Normal Men, Korean Journal of Parasitology. (2019) 57, no. 1, 21–25, 10.3347/kjp.2019.57.1.21, 30840795. 30840795 PMC6409223

[169] Tsang S. H. , Peisch S. F. , Rowan B. , Markt S. C. , Gonzalez-Feliciano A. G. , Sutcliffe S. , Platz E. A. , Mucci L. A. , and Ebot E. M. , Association Between *Trichomonas vaginalis* and Prostate Cancer Mortality, International Journal of Cancer. (2019) 144, no. 10, 2377–2380, 10.1002/ijc.31885, 30242839. 30242839 PMC6430694

[170] Brotman R. M. , Ravel J. , Bavoil P. M. , Gravitt P. E. , and Ghanem K. G. , Microbiome, Sex Hormones, and Immune Responses in the Reproductive Tract: Challenges for Vaccine Development Against Sexually Transmitted Infections, Vaccine. (2014) 32, no. 14, 1543–1552, 10.1016/j.vaccine.2013.10.010, 24135572. 24135572 PMC3964794

[171] Yadav S. , Verma V. , Singh Dhanda R. , and Yadav M. , Insights Into the Toll-Like Receptors in Sexually Transmitted Infections, Scandinavian Journal of Immunology. (2021) 93, no. 1, e12954, 10.1111/sji.12954, 32762084. 32762084

[172] Grivennikov S. I. , Greten F. R. , and Karin M. , Immunity, Inflammation, and Cancer, Cell. (2010) 140, no. 6, 883–899, 10.1016/j.cell.2010.01.025, 20303878. 20303878 PMC2866629

[173] Coussens L. M. and Werb Z. , Inflammation and Cancer, Nature. (2002) 420, no. 6917, 860–867, 10.1038/nature01322, 12490959. 12490959 PMC2803035

[174] Kay J. , Thadhani E. , Samson L. , and Engelward B. , Inflammation-Induced DNA Damage, Mutations and Cancer, DNA Repair. (2019) 83, 102673, 10.1016/j.dnarep.2019.102673, 31387777. 31387777 PMC6801086

[175] Cucchiara V. , Yang J. C. , Mirone V. , Gao A. C. , Rosenfeld M. G. , and Evans C. P. , Epigenomic Regulation of Androgen Receptor Signaling: Potential Role in Prostate Cancer Therapy, Cancers. (2017) 9, no. 1, 10.3390/cancers9010009, 28275218. PMC5295780 28275218

[176] Sharma U. , Sahu A. , Shekhar H. , Sharma B. , Haque S. , Kaur D. , Tuli H. S. , Mishra A. , and Ahmad F. , The Heat of the Battle: Inflammation′s Role in Prostate Cancer Development and Inflammation-Targeted Therapies, Discover Oncology. (2025) 16, no. 1, 10.1007/s12672-025-01829-4, 39891849. PMC11787145 39891849

[177] de Visser K. E. and Joyce J. A. , The Evolving Tumor Microenvironment: From Cancer Initiation to Metastatic Outgrowth, Cancer Cell. (2023) 41, no. 3, 374–403, 10.1016/j.ccell.2023.02.016, 36917948. 36917948

[178] Archer M. , Dogra N. , and Kyprianou N. , Inflammation as a Driver of Prostate Cancer Metastasis and Therapeutic Resistance, Cancers. (2020) 12, no. 10, 10.3390/cancers12102984, 33076397. PMC7602551 33076397

[179] Jin R. J. , Lho Y. , Connelly L. , Wang Y. , Yu X. , Saint Jean L. , Case T. C. , Ellwood-Yen K. , Sawyers C. L. , Bhowmick N. A. , Blackwell T. S. , Yull F. E. , and Matusik R. J. , The Nuclear Factor-kappaB Pathway Controls the Progression of Prostate Cancer to Androgen-Independent Growth, Cancer Research. (2008) 68, no. 16, 6762–6769, 10.1158/0008-5472.CAN-08-0107, 18701501. 18701501 PMC2840631

[180] Domingo-Domenech J. , Mellado B. , Ferrer B. , Truan D. , Codony-Servat J. , Sauleda S. , Alcover J. , Campo E. , Gascon P. , Rovira A. , Ross J. S. , Fernández P. L. , and Albanell J. , Activation of Nuclear Factor-kappaB in Human Prostate Carcinogenesis and Association to Biochemical Relapse, Br J Cancer.(2005) 93, no. 11, 1285–1294, 10.1038/sj.bjc.6602851, 16278667. 16278667 PMC2361509

[181] Al-Rashidi R. R. , Noraldeen S. A. M. , Kareem A. K. , Mahmoud A. K. , Kadhum W. R. , Ramírez-Coronel A. A. , Iswanto A. H. , Obaid R. F. , Jalil A. T. , Mustafa Y. F. , and Nabavi N. , Malignant Function of Nuclear Factor-kappaB Axis in Prostate Cancer: Molecular Interactions and Regulation by Non-Coding RNAs, Pharmacological Research. (2023) 194, 106775, 10.1016/j.phrs.2023.106775, 37075872. 37075872

[182] Zhong S. , Huang C. , Chen Z. , Chen Z. , and Luo J. L. , Targeting Inflammatory Signaling in Prostate Cancer Castration Resistance, Journal of Clinical Medicine. (2021) 10, no. 21, 10.3390/jcm10215000, 34768524. PMC8584457 34768524

[183] Shi J. , Chen J. , Serradji N. , Xu X. , Zhou H. , Ma Y. , Sun Z. , Jiang P. , du Y. , Yang J. , Dong C. , and Wang Q. , PMS1077 Sensitizes TNF-*α* Induced Apoptosis in Human Prostate Cancer Cells by Blocking NF-*κ*B Signaling Pathway, PLoS One. (2013) 8, no. 4, e61132, 10.1371/journal.pone.0061132, 23593410. 23593410 PMC3621893

[184] Mujtaba T. and Dou Q. P. , Advances in the Understanding of Mechanisms and Therapeutic Use of Bortezomib, Discovery Medicine. (2011) 12, no. 67, 471–480. 22204764 PMC4139918

[185] Pavan A. R. , Silva G. D. , Jornada D. H. , Chiba D. E. , Fernandes G. F. , Man Chin C. , and dos Santos J. , Unraveling the Anticancer Effect of Curcumin and Resveratrol, Nutrients. (2016) 8, no. 11, 10.3390/nu8110628, 27834913. PMC5133053 27834913

[186] Kwan H. Y. , Liu B. , Huang C. , Fatima S. , Su T. , Zhao X. , Ho A. H. M. , Han Q. , Hu X. , Gong R. H. , Chen M. , Wong H. L. X. , and Bian Z. , Signal Transducer and Activator of Transcription-3 Drives the High-Fat Diet-Associated Prostate Cancer Growth, Cell Death & Disease. (2019) 10, no. 9, 10.1038/s41419-019-1842-4, 31474764. PMC6717738 31474764

[187] Hellsten R. , Stiehm A. , Palominos M. , Persson M. , and Bjartell A. , The STAT3 Inhibitor GPB730 Enhances the Sensitivity to Enzalutamide in Prostate Cancer Cells, Translational Oncology. (2022) 24, 101495, 10.1016/j.tranon.2022.101495, 35917644. 35917644 PMC9344336

[188] Bishop J. L. , Thaper D. , and Zoubeidi A. , The Multifaceted Roles of STAT3 Signaling in the Progression of Prostate Cancer, Cancers. (2014) 6, no. 2, 829–859, 10.3390/cancers6020829, 24722453. 24722453 PMC4074806

[189] Tse C. , Warner A. , Farook R. , and Cronin J. G. , Phytochemical Targeting of STAT3 Orchestrated Lipid Metabolism in Therapy-Resistant Cancers, Biomolecules. (2020) 10, no. 8, 10.3390/biom10081118, 32731620. PMC7464013 32731620

[190] Kotha A. , Sekharam M. , Cilenti L. , Siddiquee K. , Khaled A. , Zervos A. S. , Carter B. , Turkson J. , and Jove R. , Resveratrol Inhibits Src and Stat3 Signaling and Induces the Apoptosis of Malignant Cells Containing Activated Stat3 Protein, Molecular Cancer Therapeutics. (2006) 5, no. 3, 621–629, 10.1158/1535-7163.MCT-05-0268, 16546976. 16546976

[191] Agarwal C. , Tyagi A. , Kaur M. , and Agarwal R. , Silibinin Inhibits Constitutive Activation of Stat3, and Causes Caspase Activation and Apoptotic Death of Human Prostate Carcinoma DU145 Cells, Carcinogenesis. (2007) 28, no. 7, 1463–1470, 10.1093/carcin/bgm042, 17341659. 17341659

[192] Takahashi T. , Uehara H. , Bando Y. , and Izumi K. , Soluble EP2 Neutralizes Prostaglandin E2-Induced Cell Signaling and Inhibits Osteolytic Tumor Growth, Molecular Cancer Therapeutics. (2008) 7, no. 9, 2807–2816, 10.1158/1535-7163.MCT-08-0153, 18790761. 18790761

[193] Richie-Jannetta R. , Nirodi C. S. , Crews B. C. , Woodward D. F. , Wang J. W. , Duff P. T. , and Marnett L. J. , Structural Determinants for Calcium Mobilization by Prostaglandin E2 and Prostaglandin F2alpha Glyceryl Esters in RAW 264.7 Cells and H1819 Cells, Prostaglandins & Other Lipid Mediators. (2010) 92, no. 1-4, 19–24, 10.1016/j.prostaglandins.2010.01.003, 20152925. 20152925 PMC3214664

[194] Xu S. , Zhou W. , Ge J. , and Zhang Z. , Prostaglandin E2 Receptor EP4 Is Involved in the Cell Growth and Invasion of Prostate Cancer via the cAMP-PKA/PI3K-Akt Signaling Pathway, Molecular Medicine Reports. (2018) 17, no. 3, 4702–4712, 10.3892/mmr.2018.8415, 29328471. 29328471

[195] Di J. M. , Zhou J. , Zhou X. L. , Gao X. , Shao C. Q. , Pang J. , Sun Q. P. , Zhang Y. , and Ruan X. X. , Cyclooxygenase-2 Expression Is Associated With Vascular Endothelial Growth Factor-C and Lymph Node Metastases in Human Prostate Cancer, Archives of Medical Research. (2009) 40, no. 4, 268–275, 10.1016/j.arcmed.2009.03.002. 19608016

[196] Klenke F. M. , Gebhard M. M. , Ewerbeck V. , Abdollahi A. , Huber P. E. , and Sckell A. , The Selective Cox-2 Inhibitor Celecoxib Suppresses Angiogenesis and Growth of Secondary Bone Tumors: An Intravital Microscopy Study in Mice, BMC Cancer. (2006) 6, no. 1, 10.1186/1471-2407-6-9, 16409625. PMC1360103 16409625

[197] Harris R. E. , Beebe J. , and Alshafie G. A. , Reduction in Cancer Risk by Selective and Nonselective Cyclooxygenase-2 (COX-2) Inhibitors, Journal of Experimental Pharmacology. (2012) 4, 91–96, 10.2147/JEP.S23826, 27186121. 27186121 PMC4863307

[198] Zappavigna S. , Cossu A. M. , Grimaldi A. , Bocchetti M. , Ferraro G. A. , Nicoletti G. F. , Filosa R. , and Caraglia M. , Anti-Inflammatory Drugs as Anticancer Agents, International Journal of Molecular Sciences. (2020) 21, no. 7, 10.3390/ijms21072605, 32283655. PMC7177823 32283655

[199] Nguyen D. P. , Li J. , and Tewari A. K. , Inflammation and Prostate Cancer: The Role of Interleukin 6 (IL-6), BJU International. (2014) 113, no. 6, 986–992, 10.1111/bju.12452. 24053309

[200] Heinrich P. C. , Behrmann I. , Haan S. , Hermanns H. M. , Müller-Newen G. , and Schaper F. , Principles of Interleukin (IL)-6-Type Cytokine Signalling and Its Regulation, Biochemical Journal. (2003) 374, no. 1, 1–20, 10.1042/bj20030407, 12773095. 12773095 PMC1223585

[201] McClelland S. , Maxwell P. J. , Branco C. , Barry S. T. , Eberlein C. , and LaBonte M. J. , Targeting IL-8 and Its Receptors in Prostate Cancer: Inflammation, Stress Response, and Treatment Resistance, Cancers. (2024) 16, no. 16, 10.3390/cancers16162797, 39199570. PMC11352248 39199570

[202] Maynard J. P. , Ertunc O. , Kulac I. , Baena-Del Valle J. A. , De Marzo A. M. , and Sfanos K. S. , IL8 Expression Is Associated With Prostate Cancer Aggressiveness and Androgen Receptor Loss in Primary and Metastatic Prostate Cancer, Molecular Cancer Research. (2020) 18, no. 1, 153–165, 10.1158/1541-7786.MCR-19-0595, 31604846. 31604846

[203] Fizazi K. , De Bono J. S. , Flechon A. , Heidenreich A. , Voog E. , Davis N. B. , Qi M. , Bandekar R. , Vermeulen J. T. , Cornfeld M. , and Hudes G. R. , Randomised Phase II Study of Siltuximab (CNTO 328), an Anti-IL-6 Monoclonal Antibody, in Combination With Mitoxantrone/Prednisone Versus Mitoxantrone/Prednisone Alone in Metastatic Castration-Resistant Prostate Cancer, European Journal of Cancer. (2012) 48, no. 1, 85–93, 10.1016/j.ejca.2011.10.014, 22129890. 22129890

[204] Bilusic M. , Heery C. R. , Collins J. M. , Donahue R. N. , Palena C. , Madan R. A. , Karzai F. , Marté J. L. , Strauss J. , Gatti-Mays M. E. , Schlom J. , and Gulley J. L. , Phase I Trial of HuMax-IL8 (BMS-986253), an Anti-IL-8 Monoclonal Antibody, in Patients With Metastatic or Unresectable Solid Tumors, Journal for ImmunoTherapy of Cancer. (2019) 7, no. 1, 10.1186/s40425-019-0706-x, 31488216. PMC6729083 31488216

[205] González-Reyes S. , Fernández J. M. , González L. O. , Aguirre A. , Suárez A. , González J. M. , Escaff S. , and Vizoso F. J. , Study of TLR3, TLR4, and TLR9 in Prostate Carcinomas and Their Association With Biochemical Recurrence, Cancer Immunology, Immunotherapy. (2011) 60, no. 2, 217–226, 10.1007/s00262-010-0931-0, 20978888. 20978888 PMC11028925

[206] Greulich B. M. , Plotnik J. P. , Jerde T. J. , and Hollenhorst P. C. , Toll-Like Receptor 4 Signaling Activates ERG Function in Prostate Cancer and Provides a Therapeutic Target, NAR Cancer. (2021) 3, no. 1, zcaa046, 10.1093/narcan/zcaa046. 33554122 PMC7848947

[207] Kundu S. D. , Lee C. , Billips B. K. , Habermacher G. M. , Zhang Q. , Liu V. , Wong L. Y. , Klumpp D. J. , and Thumbikat P. , The Toll-Like Receptor Pathway: A Novel Mechanism of Infection-Induced Carcinogenesis of Prostate Epithelial Cells, Prostate. (2008) 68, no. 2, 223–229, 10.1002/pros.20710, 18092352. 18092352

[208] Ou T. , Lilly M. , and Jiang W. , The Pathologic Role of Toll-Like Receptor 4 in Prostate Cancer, Frontiers in Immunology. (2018) 9, 10.3389/fimmu.2018.01188, 29928275. PMC5998742 29928275

[209] Gatti G. , Rivero V. , Motrich R. D. , and Maccioni M. , Prostate Epithelial Cells Can Act as Early Sensors of Infection by Up-Regulating TLR4 Expression and Proinflammatory Mediators Upon LPS Stimulation, Journal of Leukocyte Biology. (2006) 79, no. 5, 989–998, 10.1189/jlb.1005597, 16522744. 16522744

[210] Hua D. , Liu M.-y. , Cheng Z.-d. , Qin X.-j. , Zhang H.-m. , Chen Y. , Qin G. J. , Liang G. , Li J. N. , Han X. F. , and Liu D. X. , Small Interfering RNA-Directed Targeting of Toll-Like Receptor 4 Inhibits Human Prostate Cancer Cell Invasion, Survival, and Tumorigenicity, Molecular Immunology. (2009) 46, no. 15, 2876–2884, 10.1016/j.molimm.2009.06.016, 19643479. 19643479

[211] Jain S. , Suklabaidya S. , Das B. , Raghav S. K. , Batra S. K. , and Senapati S. , TLR4 Activation by Lipopolysaccharide Confers Survival Advantage to Growth Factor Deprived Prostate Cancer Cells, Prostate. (2015) 75, no. 10, 1020–1033, 10.1002/pros.22983, 25833062. 25833062

[212] Ilvesaro J. M. , Merrell M. A. , Swain T. M. , Davidson J. , Zayzafoon M. , Harris K. W. , and Selander K. S. , Toll Like Receptor-9 Agonists Stimulate Prostate Cancer Invasion In Vitro, Prostate. (2007) 67, no. 7, 774–781, 10.1002/pros.20562, 17373717. 17373717

[213] Karan D. , Tawfik O. , and Dubey S. , Expression Analysis of Inflammasome Sensors and Implication of NLRP12 Inflammasome in Prostate Cancer, Scientific Reports. (2017) 7, no. 1, 10.1038/s41598-017-04286-4, 28663562. PMC5491527 28663562

[214] Xu Z. , Wang H. , Qin Z. , Zhao F. , Zhou L. , Xu L. , and Jia R. , NLRP3 Inflammasome Promoted the Malignant Progression of Prostate Cancer via the Activation of Caspase-1, Cell Death Discovery. (2021) 7, no. 1, 10.1038/s41420-021-00766-9, 34930938. PMC8688424 34930938

[215] Shadab A. , Mahjoor M. , Abbasi-Kolli M. , Afkhami H. , Moeinian P. , and Safdarian A. R. , Divergent Functions of NLRP3 Inflammasomes in Cancer: A Review, Cell Communication and Signaling. (2023) 21, no. 1, 10.1186/s12964-023-01235-9, 37715239. PMC10503066 37715239

[216] Hsu L.-C. , Enzler T. , Seita J. , Timmer A. M. , Lee C.-Y. , Lai T.-Y. , Yu G. Y. , Lai L. C. , Temkin V. , Sinzig U. , Aung T. , Nizet V. , Weissman I. L. , and Karin M. , IL-1*β*-Driven Neutrophilia Preserves Antibacterial Defense in the Absence of the Kinase IKK*β* , Nature Immunology. (2011) 12, no. 2, 144–150, 10.1038/ni.1976, 21170027. 21170027 PMC3677078

[217] Panchanathan R. , Liu H. , and Choubey D. , Hypoxia Primes Human Normal Prostate Epithelial Cells and Cancer Cell Lines for the NLRP3 and AIM2 Inflammasome Activation, Oncotarget. (2016) 7, no. 19, 28183–28194, 10.18632/oncotarget.8594, 27058421. 27058421 PMC5053719

[218] Pérez-Gómez J. M. , Montero-Hidalgo A. J. , Fuentes-Fayos A. C. , Sarmento-Cabral A. , Guzmán-Ruiz R. , Malagón M. M. , Herrera-Martínez A. D. , Gahete M. D. , and Luque R. M. , Exploring the Role of the Inflammasomes on Prostate Cancer: Interplay With Obesity, Reviews in Endocrine and Metabolic Disorders. (2023) 24, no. 6, 1165–1187, 10.1007/s11154-023-09838-w, 37819510. 37819510 PMC10697898

[219] Zhang L. G. , Chen J. , Meng J. L. , Zhang Y. , Liu Y. , Zhan C. S. , Chen X. G. , Zhang L. , and Liang C. Z. , Effect of Alcohol on Chronic Pelvic Pain and Prostatic Inflammation in a Mouse Model of Experimental Autoimmune Prostatitis, Prostate. (2019) 79, no. 12, 1466–1476, 10.1002/pros.23866. 31233226

